# Maturation of the Autonomic Nervous System in Premature Infants: Estimating Development Based on Heart-Rate Variability Analysis

**DOI:** 10.3389/fphys.2020.581250

**Published:** 2021-01-12

**Authors:** Mario Lavanga, Elisabeth Heremans, Jonathan Moeyersons, Bieke Bollen, Katrien Jansen, Els Ortibus, Gunnar Naulaers, Sabine Van Huffel, Alexander Caicedo

**Affiliations:** ^1^Division STADIUS, Department of Electrical Engineering (ESAT), Katholieke Universiteit Leuven, Leuven, Belgium; ^2^Department of Development and Regeneration, Faculty of Medicine, Katholieke Universiteit Leuven, Leuven, Belgium; ^3^Applied Mathematics and Computer Science, School of Engineering, Science and Technology, Universidad del Rosario, Bogotá, Colombia

**Keywords:** preterm infants, HRV, bradycardia, autonomic nervous system, development

## Abstract

This study aims at investigating the development of premature infants' autonomic nervous system (ANS) based on a quantitative analysis of the heart-rate variability (HRV) with a variety of novel features. Additionally, the role of heart-rate drops, known as bradycardias, has been studied in relation to both clinical and novel sympathovagal indices. ECG data were measured for at least 3 h in 25 preterm infants (gestational age ≤32 weeks) for a total number of 74 recordings. The post-menstrual age (PMA) of each patient was estimated from the RR interval time-series by means of multivariate linear-mixed effects regression. The tachograms were segmented based on bradycardias in periods after, between and during bradycardias. For each of those epochs, a set of temporal, spectral and fractal indices were included in the regression model. The best performing model has *R*^2^ = 0.75 and mean absolute error *MAE* = 1.56 weeks. Three main novelties can be reported. First, the obtained maturation models based on HRV have comparable performance to other development models. Second, the selected features for age estimation show a predominance of power and fractal features in the very-low- and low-frequency bands in explaining the infants' sympathovagal development from 27 PMA weeks until 40 PMA weeks. Third, bradycardias might disrupt the relationship between common temporal indices of the tachogram and the age of the infant and the interpretation of sympathovagal indices. This approach might provide a novel overview of post-natal autonomic maturation and an alternative development index to other electrophysiological data analysis.

## 1. Introduction

Premature infants represent 10% of the neonatal population and are at higher risk for developmental disorders that can lead to adverse outcome (Aylward, 2014). The investigation of maturation via multiple physiological biomarkers is part of the clinical practice to prevent lower cognitive, motor, or language outcomes later on in life (Franke et al., 2012; Koolen et al., 2016). A common probe to inspect the development of the neurovegetative functions or *Autonomic Nervous System* (ANS) is the heart-rate fluctuation, simply known as *Heart-rate variability* (HRV).

The guidelines of the adult HRV task force clearly specify the association between the different frequency tones of the tachograms and the stimulation of the ANS branches (Camm et al., 1996). The stimulation of the sympathetic branch is normally represented by the low-frequency band (*LF*, [0.04−0.15] *Hz*) of the HRV, while the high-frequency band (*HF*, [0.15−0.4] *Hz*) reflects the parasympathetic branch. The sympathovagal balance can be expressed by the power ratio of the two frequency bands $\left(\frac{LF}{HF}\right)$, while the very-low-frequency band (*VLF*, [0−0.04] *Hz*) is usually associated to thermal and hormonal regulation. On the contrary, the fetal and preterm HRV frequency bands are still the subject of an intensive discussion in the literature. The early exposure to the *ex-utero* environment induces an aberrant sympathetic response and delays autonomic maturation (Smith et al., 2013; Javorka et al., 2017). The association between the common HRV frequency bands and the sympathovagal regulation is far less documented in infants and fetuses (Doret et al., 2015). Other factors are known to play a role in the definition of the oscillations of the heart rate, such as intermittent breathing cycles with high respiratory frequency and the actual delay in maturation of the autonomic nervous system. Therefore, David et al. (2007), Hoyer et al. (2013), and Doret et al. (2015) suggested that new ways to investigate the sympathovagal balance should be examined. Since the fetal heart-rate is characterized by a strong slow-wave baseline, David et al. redefine the frequency bands for fetuses as follows: *VLF* = [0.02−0.08] *Hz*, *LF* = [0.08−0.2] *Hz*, *HF* = [0.2−3] *Hz*. While adults normally present an HRV spectrum with two clear peaks at *HF* and *LF* (Camm et al., 1996), infants and fetuses have a 1/*f* spectrum up to 0.1 *Hz* (Karin et al., 1993). Consequently, the full description of the preterm ANS has to consider all the possible frequency bands (*VLF*,*LF*,*HF*) (Clairambault et al., 1992; Curzi-Dascalova, 1994; Mazursky et al., 1998; Longin et al., 2006). This could explain why the $\frac{LF}{HF}$ ratio can give contradictory results: Krueger et al. (2010) did not find any specific change in this ratio in a longitudinal study with preterm patients, while Longin et al. (2006) found a decrease in $\frac{LF}{HF}$ from preterm to term age. The rapid development and the unclear definition of the sympathovagal frequency bands might not give a simple interpretation of $\frac{LF}{HF}$ as it is for adults. Surprisingly, infants show greater changes in the absolute power of the three main bands *VLF*, *LF* and *HF* than relative power (Longin et al., 2006). Hoyer et al. (2013) argued that predominant principles of autonomic development are not only an increase in heart-rate variability but also an increase in the complexity and pattern formation. Consequently, HRV indices can be chosen to reflect these principles in order to describe the sympathovagal balance maturation. Pattern formation can be described by tachogram skewness and the new ratio $\frac{VLF}{LF}$, while the increasing complexity is characterized by an increasing HRV entropy. It should also be stressed that the computation of power ratios, such as $\frac{LF}{HF}$, requires stationarity, which can be questioned in the case of infants heart-rate time series. Therefore, Abry et al. (2010) and Doret et al. (2015) proposed fractal analysis as an alternative method to investigate the sympathovagal balance in fetal heart-rate. It focuses on quantities, such as oscillations or increments at different scales to tackle the absence of stationarity and determines specific relations between the fractal exponents (such as the Hurst Exponent) and the $\frac{LF}{HF}$ ratios. However, those methods were never applied to premature infants.

One example of non-stationarity is the presence of bradycardias. These are normally heart-rate drops below 70% of the heart-rate average, which last at least for 4 s and may be associated with apneas or hypoxias (Poets et al., 1993). These drops can alter oxygen saturation and blood flow, putting organs at risk of damage (Paolillo and Picone, 2013). Apneic spells that occur with bradycardias or hypoxic events are most likely to affect brain homeostasis. In addition, those physiological instabilities are the probable consequence of the immature respiratory system (Porges, 1995; Atkinson and Fenton, 2009). However, it has also been shown different HR reactions can be triggered by hyperoxia and hypoxias via chemoreception in term infants (Søvik et al., 2001). Additionally, Poets et al. specifically highlighted that bradycardias can occur independently from apneic or gas events (Poets et al., 1993). Bradycardias can be considered a consequence of heart-rate dysregulation, which can disrupt the state-space and the probability density function of the tachogram (Gee et al., 2017). Any proper model that tries to describe the development of the infants' ANS has to include not simply the slow variation of the basal heart-rate, but the sudden drops of the tachogram, independently from any other conditioning factor. Those non-stationary events possibly affect the most common HRV temporal or spectral features used in clinical practice, such as the standard deviation and the $\frac{LF}{HF}$ ratio. They are commonly used for the assessment of development outcome, sleep or pathologies diagnosis (Javorka et al., 2017), and any disruption of these features can bias conclusions made by the medical community. For example, bradycardias can forcefully increase the variability of the tachogram or its regularity.

In order to address the shortcomings using the studies outlined above and the lack of autonomic growth charts for premature infants, a new framework to describe autonomic maturation in healthy preterm babies has been provided. This research can be divided into two main strands. First, both spectral analysis and multifractal analysis have been employed to investigate the neurovegetative development of the sympathovagal balance and its complexity and track maturation. Second, the impact of bradycardias on both clinical and novel ANS maturation indices has been investigated. This study tries to provide a complete overview of autonomic maturation in premature neonates, including non-stationary events, such as bradycardias. The final clinical objective is to provide novel maturation charts for the premature autonomic nervous system in the first weeks of life and correct the effect of heart-rate events on common clinical HRV indices. Those normative charts might be used as references to investigate early-life and *ex-utero* factors that can deviate from normal premature development and define suitable therapies in the neonatal intensive care unit.

## 2. Methods

### 2.1. Dataset

The dataset consists of electrocardiograms (ECG) of 25 preterm infants, which were recorded at the Neonatal Intensive Care Unit of the University Hospital of Leuven. It was collected in a multimodal setting for another research study related to brain development and a sleep-stage analysis (Koolen et al., 2016; Dereymaeker et al., 2017). Inclusion criteria were as follows: a normal neurodevelopmental outcome at 9 and 24 months corrected age (Bayley Scales of Infant Development-II, mental and motor score >85), no severe brain lesions, assessed by ultrasound and not taking any sedative or antiepileptic drugs during the EEG registration. The sampling ECG frequency was 250 or 500 *Hz* and the average length of the recording was 4 h 44 min. An overview of the dataset is reported in Table 1, while a complete description is reported in Supplementary Tables 1, 2. The latter shows the heterogenous interperiod sessions among recordings, which indicate that the measurements were not scheduled at the same PMA for each patient. In addition, the Supplementary Materials 1, 2 show the ECG sampling frequency for each of the recording.

**Table 1:** Demographics of the 25 patients: average duration of the tachogram in minutes (Duration_*Rec*_), average duration of the annotated bradycardias in s (Duration_*WB*_), average number of the annotated bradycardias (Number_*WB*_), average RR amplitude during the bradycardia in ms (RR_*WB*_), post-menstrual age in weeks (PMA) and gestational age in weeks (GA).

**Number of patients = 25**	
Duration_*Rec*_ (min)	208.435 ± 115.657
Duration_*WB*_ (s)	18.881 ± 8.332
Number_*WB*_	7 ± 12
RR_*WB*_	631.405 ± 78.677
PMA (*wks*)	33.689 ± 3.049
GA (*wks*)	28.315 ± 2.318
*PMA* ≤ 32 *wks*	22
*PMA* ∈ (32−36] *wks*	35
*PMA* > 36 *wks*	17

The last three rows represent the number of recording for each age subgroup (below 32 PMA weeks, between 32 and 36 weeks, and above 36 weeks).

### 2.2. Pre-processing

The HRV represents the instantaneous fluctuations of heart rate and is usually expressed by the tachogram which visualizes the variations of the time interval between two consecutive R-peaks (RR intervals, *RR*_*i*_). In order to compute a *RR*_*i*_ time series, the R peaks of the ECG have been detected via the Matlab toolbox by Moeyersons et al. (2019), which is based on enveloping procedure. This graphical user-interface also allows for correction and deletion in case of erroneous R-peaks. In case of a single missing R-peak, the value was replaced by using the following formula:

(1) $$\begin{array}{l} {\hat{R}}_{t}=\frac{R_{t-1}+R_{t+1}}{2}, \end{array}$$

where ${\hat{R}}_{t}$ is the estimated position of the missed R-peak, while *R*_*t*−1_ and *R*_*t*+1_ are the location of the previous and following R-peak. In the case of two or more missing R-peaks due to ECG flat lines or muscle artifacts, which made the QRS detection impossible, the contaminated parts of the signal were discarded. In case that less 20 min of noise-free signal remained, the signal was discarded. The length of each recording (in min) is reported in Supplementary Tables 1, 2. The progressive number of the recording ID shows that some of them were fully discarded. The full overview shows that all included recordings had at least 50 min of available data (Duration_*Rec*_ column) resulting in a total of 74 recordings.

Besides the preprocessing of artifacts and before the feature extraction, we also dealt with the sudden drops of heart-rate, known as bradycardias. Although those phenomena are completely natural in the developing infant, they can suddenly increase the frequency content of the *RR*_*i*_ series. Therefore, traditional linear spectral and temporal analysis might not be suitable since the instantaneous variance and mean of the heart-rate can vary over time, as explained in detail by Gee et al. (2017). According to the same study, the heart-rate activity that precedes sudden drops might differ from the drops itself and other bradycardia-free periods. Consequently, bradycardias have been detected in the current studies before any further processing. Based on the definitions of apnea of prematurity and bradycardias by Paolillo and Picone (2013), the bradycardia spells were detected as sudden $\bar{RR_{i}}$ increases above $\theta =1.5*\bar{RR_{i}}$ that persist for more than 4 s, where $\bar{RR_{i}}$ is the median tachogram of the entire recording. We defined the onset of the bradycardia as the moment that the tachogram exceeds θ. Conversely, the offset was defined as the moment that the amplitude decreases below the same threshold. Subsequently, three different windowing strategies were applied:

1. Post-bradycardia (PB) windowing: the 10-min period that starts 10 s after the bradycardia offset was considered a candidate for features extraction. This window did not include the bradycardia itself.
2. Between-bradycardias (BB) windowing: all non-overlapping 10-min windows contained between bradycardic events were considered as candidate epochs for features' extraction. The first viable window was at least 10 min from the bradycardia offset in order to guarantee that the signal was stabilized.
3. Within-bradycardia (WB) windowing: a 10-min window was considered from the bradycardia onset. This windowing should involve both the information related to the heart-rate drop and the recovery period. Based on the definition of the PB, the PB and WB windowing schemes overlap almost fully for the period after the bradycardia offset, but WB is the only scheme fully containing the bradycardic event.

**Figure 1:**
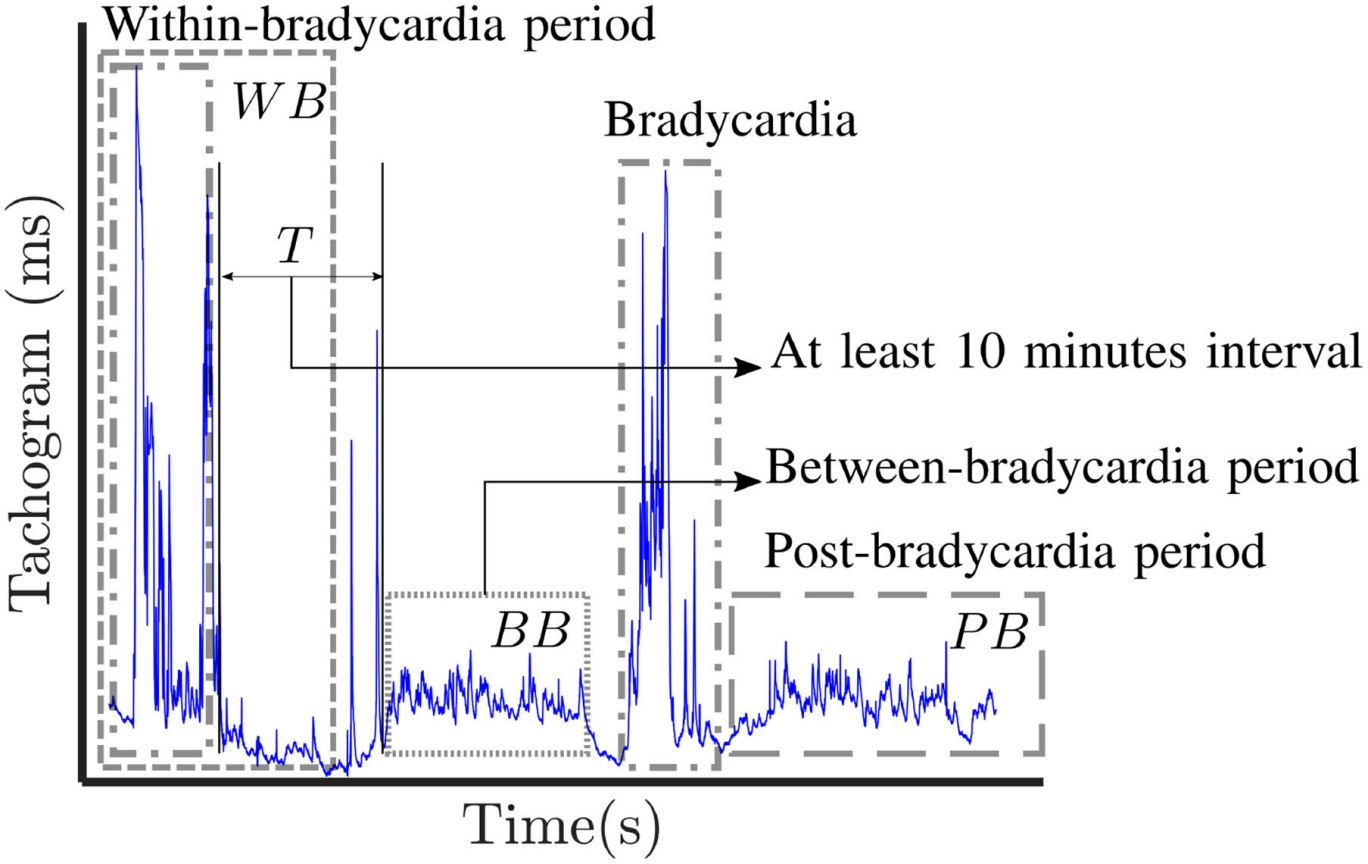
Visual representation of the three windowing schemes applied in this study: post-bradycardia scheme (*PB*), between-bradycardia scheme (*BB*) and within-bradycardia scheme (*WB*). The selected windows in each trace are indicated with gray dashed boxes, while the dot-dashed boxes show examples of annotated bradycardia. In case of *BB* windowing, a period *T* greater that 10 min is present between the end of the bradycardia and the first available window.

**Figure 2:**
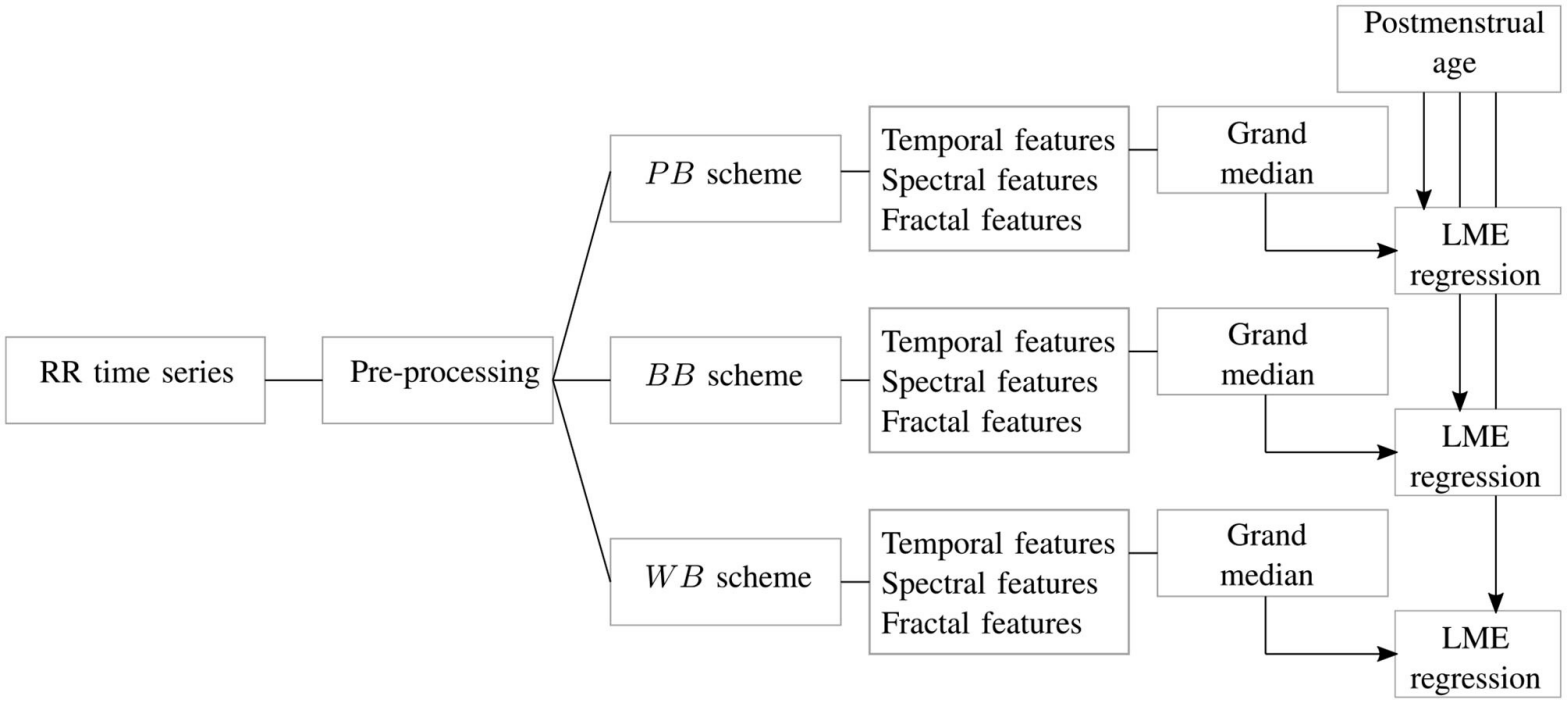
The block diagram shows the main steps of the study. For each RR signal, artifact preprocessing is performed and associated resampling of the tachogram. The signal is split in different windows according to the scheme of Figure 1. For each of these epochs, temporal, spectral and fractal features undergo a grand-median process if there is more than one epoch per scheme. The three datasets are then used to estimate the age of the recording in a linear mixed effects (LME) regression.

A visual description of the windowing scheme is reported in Figure 1. The gray dashed boxes highlight the three types of windows (*WB*, *BB*, *PB*) that can be determined in a single trace, while the dot-dash box shows typical bradycardia events. The average duration and amplitude of a bradycardia event are reported in Table 1, which also shows the average number of bradycardias in the entire dataset. A full overview recording by recording is reported in the Supplementary Tables 1, 2, which display the number of bradycardias as well as the average intensity and the average duration of heart-rate drops patient by patient. The Supplementary Material shows that some of the recordings did not have any heart-rate drop according to the reported definition. Therefore, the windowing scheme based on bradycardias was not applicable. In this specific case, the design choice was a segmentation in non-overlapping 10-min windows and assign the results of feature extraction to post-bradycardia windowing scheme (see Figure 2).

### 2.3. Feature Extraction

In each of the windows defined according to the *PB*, *BB*, and *WB* schemes, a set of temporal, spectral and fractal features were derived to describe the autonomic nervous system of the premature infants and its relationship with development. These features were chosen based on the principles of variability increase, complexity increase and pattern formation by Hoyer et al. (2013). An overview of the different attributes is reported in Table 2.

**Table 2:** Overview of all computed features.

**Temporal features**		
Statistical moments		μ_*RR*_, σ_*RR*_
	**Spectral features**	
Welch		*P*(*VLF*),*P*(*LF*),*P*(*HF*),
		$\frac{VLF}{LF}$,$\frac{LF}{HF}$,$\frac{LF}{LF+HF}$,$\frac{LF}{LF+VLF}$
SPWVD		*P*(*VLF*),*P*(*LF*),*P*(*HF*),
		$\frac{VLF}{LF}$,$\frac{LF}{HF}$,$\frac{LF}{LF+HF}$,$\frac{LF}{LF+VLF}$
Wavelet		*P*(*VLF*),*P*(*LF*),*P*(*HF*),
		$\frac{VLF}{LF}$,$\frac{LF}{HF}$,$\frac{LF}{LF+HF}$,$\frac{LF}{LF+VLF}$
	**Fractal features**	
Multifractality		*H*_*exp*, [*j*_1_, *j*_2_ = **5**, 12]_, *C*_2, [*j*_1_, *j*_2_ = **5**, 12]_,
		*H*_*exp*, [*j*_1_, *j*_2_ = 3, 12]_,*C*_2, [*j*_1_, *j*_2_ = 3, 12]_

Besides the first- and the second-order moments, the table reports all the relative and absolute power features in the frequency domain, namely the power in VLF, LF, and HF bands, the ratio between the VLF and LF bands and between LF and HF bands and the normalized LF band power with respect to VLF band and HF band. The spectral features are reported for each the PSD estimation approaches that were used in this study. The last section reports the computed multifractal features, i.e., Hurst exponent (H_exp_) and the (C_2_) for the investigated scale ranges: [j_1_,j_2_]=[3,12] and [j_1_,j_2_]=[5,12].

#### 2.3.1. Temporal Indices

Based on the most common guidelines related to HRV processing (Camm et al., 1996; Javorka et al., 2017), the first- and the second-order moments of the *RR*_*i*_, i.e., the mean of the tachogram (μ_*RR*_) and the standard deviation (σ_*RR*_), were computed in order to assess the level of the variability.

#### 2.3.2. Spectral Analysis

The sympathovagal activity is normally assessed by the computation of the spectral power in the different HRV frequency bands (Camm et al., 1996). Unlike adults, premature infants have a higher mean heart rate with very slow oscillation around it (Clairambault et al., 1992; David et al., 2007). Therefore, the frequency bands of the premature patients were defined as follows: *VLF* = [0, 0.08] *Hz*, the low-frequency *LF* = [0.08, 0.2] *Hz* and high frequency *HF* = [0.2, 3.0] *Hz*. Additionally, the RR time series of the premature infant can be non-stationary due to a series of events, like bradycardias or other heart-rate dysregulation. Therefore, the power spectral density was computed with time-frequency (TF) methodologies, which allows us to investigate the principle of pattern formation as discussed by Hoyer et al. (2013). Namely, the HRV power spectral density (PSD) was estimated with three specific approaches: the Welch's periodogram, the quadratic smoothed pseudo Wigner-Ville distribution (SPWD) (Orini et al., 2012) and the continuous wavelet transform (CWT) (David et al., 2007).

Given a fixed window size, Welch's algorithm estimates multiple periodograms in overlapping subwindows and averages them. The Welch's window length was set at 3 min and the overlap at 50%. Based on the suggestions of the HRV guidelines (Camm et al., 1996), the window length was set to investigate the very-low-frequency band.

One can also estimate the instantaneous autospectrum *S*_*RR*_(*t, f*). Based on the CWT of the tachogram

(2) $$\begin{array}{l} W_{RR}(t,s)=\int_{-\infty }^{+\infty }RR(\tau )\psi^{*}(\frac{t-\tau }{s})d\tau , \end{array}$$

*S*_*RR*_(*t, f*) can be computed as the scalogram of the wavelet transform of the signal as follows:

(3) $$\begin{array}{l} S_{RR}\left(t,f\right)=|W_{RR}\left(t,f\right){|}^{2}, \end{array}$$

where ψ is the mother wavelet (Analytic Morlet), while *s* stands for the scale of the wavelet transform and, in the general, *s* ≈ *f*^−1^. However, the *S*_*RR*_(*t, f*) based on CWT risks to be distorted by interference terms which can be present with linear time-frequency approaches. Therefore, Orini et al. (2012) proposed to estimate the instantaneous autospectrum via a quadratic time-frequency distribution, such as SPWD. Then *S*_*RR*_ is then estimated as follows:

(4) $$\begin{array}{l} S_{RR}\left(t,f\right)=\int \int_{-\infty }^{+\infty }\Phi_{RR}\left(\tau ,\nu \right)A_{RR}\left(\tau ,\nu \right)e^{j2\pi \left(t\nu -\tau f\right)}d\tau d\nu , \end{array}$$

where *A*_*RR*_(τ, ν) is the ambiguity function, which is defined as the Fourier Transform of the time-dependent auto-correlation of *RR*(*t*) as follows

(5) $$\begin{array}{l} A_{RR}\left(\tau ,\nu \right)=\int_{-\infty }^{+\infty }RR\left(t+\tau /2\right)RR^{*}\left(t-\tau /2\right)e^{-j2\pi t\nu }dt, \end{array}$$

The smoothing of the time-frequency cross-coupling in (4) is done via the exponential kernel in the ambiguity domain defined as

(6) $$\begin{array}{l} \Phi_{RR}(\tau ,\nu )=\exp\left\{-\pi {\left[{\left(\frac{\nu }{\nu_{0}}\right)}^{2}+{\left(\frac{\tau }{\tau_{0}}\right)}^{2}\right]}^{2\lambda }\right\}, \end{array}$$

Following the study by Widjaja et al. (2013), ν_0_, τ_0_, λ were set to 0.050, 0.046, and 0.3, respectively, leading to a kernel function with a TF resolution of [Δ*t*, Δ*f*] = [10.9 s, 0.03 Hz]. Both Welch's approach and the CWT were computed with MATLAB subroutines, while the implementation details and software download for the SPWD are reported in Orini et al. (2012).

Based on the given methodologies, the instantaneous power in the β = [*f*_1_, *f*_2_] band of interest can be obtained as

(7) $$\begin{array}{l} P_{\beta }\left(t\right)=\int_{f1}^{f2}S_{RR}\left(t,f\right)df. \end{array}$$

In particular, one can compute the absolute power in three bands *VLF*, *LF* and *HF* as the reported integral in (7). Besides, the ratios $\frac{VLF}{LF}$ and $\frac{LF}{HF}$ were also computed alongside two indices to represent the normalized *LF* power: $\frac{LF}{VLF+LF}$ and $\frac{LF}{HF+LF}$. In case of Welch's algorithm, there is no dependency from the time variable *t*. On the contrary, CWT and SPWD generate a time series for each selected frequency band, as highlighted by (3), (4), and (7). In order to obtain one single value for each window, the median of this time-series was taken into account. The set of spectral features derived for each methodology is reported in the central block of Table 2.

#### 2.3.3. Multifractal Analysis

Since spectral analysis requires stationarity of data and the very definition of the tachogram series frequency bands have been questioned, the HRV was also analyzed according to the fractal or multifractal paradigm. The multifractal analysis aimed to describe the principle of complexity increase discussed by Hoyer et al. (2013). As shown in Doret et al. (2015), the infant's tachogram is a fractal or scale-free signal, which presents a power-law decay spectrum as follows:

(8) $$\begin{array}{l} S_{RR}\left(f\right)=|C|f^{-2\left(H_{exp}-1\right)} \end{array}$$

where *H*_*exp*_ is known as the Hurst exponent and controls the decay of the power function. H is also a representative parameter for fractal time series and there can be more than one exponent for each signal. A signal with one single exponent is commonly known as monofractal, while a signal with multiple exponents *h* is known as multifractal (Ivanov et al., 1999). Small values of *h* represent sharp and transient regularity or singularity, while large values represent smooth changes (Leonarduzzi et al., 2010).

An efficient method to determine the amount of exponents or singularities *h* is the multifractal formalism based on the wavelet transform modulus maxima. The discrete wavelet transform (DWT) decomposes the signal *RR*(*t*) into elementary time-frequency components based on different scales *a*. Large scales describe smooth and low frequency oscillations, while small scales describe the sharp transitions in the signal. According to Popivanov et al. (2006) and Wendt et al. (2007), a partition function $Z\left(a,q\right)=Z_{L}\left(2^{j},q\right)$ can be estimated using the *wavelet leader* *L*_*f*_(*j, k*), as follows:

(9) $$\begin{array}{l} Z\left(a,q\right)=Z_{L}\left(2^{j},q\right)=\frac{1}{n_{k}}\overset{n_{k}}{\underset{k=1}{\sum }}|L_{f}\left(j,k\right){|}^{q}\sim 2^{j\tau \left(q\right)}, \end{array}$$

where *L*_*f*_(*j, k*) represents a specific type of wavelet transform, where the maximum of wavelet coefficients is considered in a narrow time neighborhood. More details can be found in Wendt et al. (2007) and Abry et al. (2010).

One can immediately notice the similarity between (8) and (9), especially between the Hurst exponents and τ(*q*). For certain values of *q*, the scaling exponent τ(*q*) (SE) has a specific meaning: for positive, respectively negative *q*, *Z*(*a, q*) reflects scaling of large, respectively short fluctuations. In general, for each *q*, the partition function exhibits a power-law decay characteristic, such as the power spectrum of 1/*f* noise (8). The scaling exponents τ(*q*) (SE) associated with this decay can be obtained by computing the slope of *Z* vs. the scales in a log-log diagram from a certain scale $a_{1}=2^{j_{1}}$ to a certain scale $a_{2}=2^{j_{2}}$. The log-transform clearly shows the advantage to define scales as power quantities. In case of a monofractal signal, τ(*q*) is a linear function of q and *H*_*exp*_, which is τ(*q*) = *qH*_*exp*_−1 (as also shown in 8). In case of a multifractal signal, the τ(*q*) is a non-linear function of the local exponents *h* and its fractal dimensions *D*(*h*), known also as the singularity spectrum (SS) (Popivanov et al., 2006).

The fractal paradigm fully describes the properties of the signal by means of the singularity spectrum *D*(*h*), obtained as the Legendre transform of τ(*q*) (Abry et al., 2010). This function represents the embedding dimensions in the function of the different singularities of the signals and two main *D*(*h*) attributes are normally derived: the maximum and the width of the *D*(*h*) distribution, known also as the parameters *C*_1_ and *C*_2_. They can be computed as cumulants or coefficients of the Taylor expansion of τ(*q*) and they are used to represent the main Hurst exponent of the multifractal signal (*H*_*exp*_ or *C*_1_) and the “variety” of fractals in the time series (*C*_2_).

The multifractality parameters (*H*_*exp*_, *C*_2_) were computed in the entire non-overlapping window according to three schemes discussed in section 2.2. Specifically, the multifractal features were derived using the Wavelet p-Leader and Bootstrap based MultiFractal analysis (PLBMF) MATLAB toolbox, described in Wendt et al. (2007). This toolbox can be downloaded from https://www.irit.fr/~Herwig.Wendt/software.html. A fundamental design choice is the scale range $\left[2^{j_{1}},2^{j_{2}}\right]$ from which the exponent τ(*q*) is estimated from (9) (Wendt et al., 2007; Abry et al., 2010). In case of HRV, the exponents [*j*_1_, *j*_2_] are normally set equal to [3,12]. Given the fact that the scale can be written as $a=2^{j}=\left(f_{s}/2\right)/f$ with *f*_*s*_ as sampling frequency of the signal, it follows that the range [*j*_1_, *j*_2_] = [3,12] approximately represents the frequency band ≈ [0.375, 0.001] *Hz* with *f*_*s*_ = 6 *Hz*. In case that [*j*_1_,*j*_2_]=[5,12], the chosen scale range approximately represents the frequency band ≈ [0.094, 0.001] *Hz*. It is clear the first range considers part of the *HF* band, while the latter solely focuses on the combination of *LF* and *VLF*. A window size of 10 min does not allow an investigation of oscillations lower than up to 0.001 Hz. However, since the VLF band of infants is limited to 0.01 Hz, we can state that the scale range [*j*_1_,*j*_2_]=[5,12] fully describes slow-waves of infants HRV. Since the chosen scale range might influence the multifractal attributes, both ranges were tested in this study to investigate which frequency bands mostly reflects the sympathovagal balance. In fact, the main Hurst exponent *H*_*exp*_ or *C*_1_ parameter is able to influence the ratio $\frac{LF}{HF}$. Based on (8), one can rewrite the spectral ratios as follows:

(10) $$\begin{array}{l} \frac{LF}{HF}=\frac{\int_{f_{m}}^{f_{I}}S_{RR}\left(f\right)df}{\int_{f_{I}}^{f_{M}}S_{RR}\left(f\right)df}=\frac{\left(f_{I}^{2-2H_{exp}}-f_{m}^{2-2H_{exp}}\right)}{\left(f_{M}^{2-2H_{exp}}-f_{I}^{2-2H_{exp}}\right)} \end{array}$$

where [*f*_*m*_, *f*_*I*_],[*f*_*I*_, *f*_*M*_] represent the frequency bands of *LF* and *HF*. Taking into account that the Hurst exponent and the $\frac{LF}{HF}$ are related and taking also into account that the chosen [*j*_1_, *j*_2_] decides which frequency bands the multifractal parameters are related to, the scales investigation of the fractal properties can shed a light which bands mainly reflect the sympathovagal activity. The set of fractal features derived for each methodology is reported in the last block of Table 2.

#### 2.3.4. Algorithmic Pipeline and Statistical Analysis

The processing pipeline of the current study is reported in Figure 2. For each HR time series, the signal was split according to the *PB*, *BB*, and *WB* windowing scheme reported in Figure 1 and all the features reported in Table 2 were extracted. Besides artifact removal, a fundamental preprocessing step is the resampling of the tachogram. The behavior of non-linear features can depend on the sample rate, as also shown by the definition of the scales and their range for the multifractal parameters (section 2.3.3). Based on the findings by Bolea et al. (2016), the following sampling frequencies were tested for fractal indices: [6, 8, 12] *Hz*. In contrast, the sampling frequencies for the spectral and temporal indices was set to 6 *Hz* in order to include the higher respiratory frequency of premature infants (Camm et al., 1996; Javorka et al., 2017). The data were resampled with linear interpolation. The results are reported for only one sampling frequency in the main manuscript; however, a more complete discussion of the use of different sampling frequencies and the associated results are included in the Supplementary Material.

As described in section 2.3, the tuning and design parameters for the spectral and fractal analysis were chosen in accordance with the absence of stationarity and the persistent slow-wave baseline of the premature HRV signal. The necessity to investigate long-range fluctuations and a recovery period after events, such as bradycardias justify the segmentation in 10 min. Normally, time-frequency approaches use windows longer than 600 s to describe evolution in HRV spectrum (Orini et al., 2012; Widjaja et al., 2013) and the fractal indices also require windows of this size to fully investigate changes in regularity (Abry et al., 2010; Doret et al., 2015). Additionally, the *BB* and *WB* schemes can generate a set of windows and therefore an array of features based on the number of bradycardias present in each recording (on average, seven bradycardias per recording, as reported by Table 1). In order to obtain one representative value for each recording in each windowing scheme, the median of this array of attributes over the different windows was computed for each recording, as highlighted by the grand-median block in Figure 2.

After the features' extraction process and the grand-median step, three datasets were then obtained according to the three different windowing schemes. The number of features extracted for each dataset was then 27 in total: 21 for the spectral attributes, two for the temporal ones and four for fractal indices, as shown in Table 2.

In order to investigate the ANS maturation, the HRV features were used to estimate the PMA of the patient, as shown by the last block of the diagram in Figure 2. Since the PMA is known for each recording, a linear mixed effects (LME) regression model was developed for each dataset with PMA as response variable (Lavanga et al., 2018). The actual regression consisted of two steps. First, the features were selected via the least absolute shrinkage and selection operator (LASSO) due to the high number of features, after that the absolute power features were log-transformed. Specifically, the LASSO was repeated for 20 iterations on the entire dataset and the features which were selected in more than 40% of the total number of iterations (eight iterations out of 20) were included in the regression model (Lavanga et al., 2018). Second, a linear mixed-effect regression model was built with the selected subsets with multiple random splits of the data. In particular, the dataset was split into 70% training set and 30% test set for 20 iterations and the model was developed on the train set and tested for test set for each iteration. The performance was then assessed as *mean absolute error* *MAE* on the test set, as well as explained variance *R*^2^, both on train and test set ($R_{train}^{2}$, $R_{test}^{2}$). We also reported the *p*_*value*_ of the F-statistics for each iteration. The results were reported as median(IQR) (where IQR stands for *InterQuartile Range*) over the 20 iterations. A linear mixed effect model requires the definition of a grouping variable that introduces the random effect, and the patient ID was taken as a grouping variable since a set of one or more recordings belong to a patient [as discussed in a previous study (Lavanga et al., 2018)]. Furthermore, the LME regression with the LASSO procedure was not simply examined for the entire subset of features, but also for the three subset feature groups: temporal, spectral and fractal attributes. In case of temporal features regression, the LASSO step was not performed.

On top of that, the trend for the ANS features throughout the patients' development was also reported as median(IQR) in three age groups (PMA ≤ 31 weeks, PMA ∈(31−36] weeks, and PMA >36 weeks) as well as Pearson correlation coefficient with PMA.

## 3. Results

The overview of the dataset is reported in Table 1, which shows certain traits of the annotated bradycardias. The mean length is around 18 s. On average, there are seven bradycardias per recording and the mean *RR*_*i*_ during bradycardias is ~631 ms. The overview shows that the infants have an average PMA of 34 weeks and 35 recordings are collected in the range (32–36] weeks. A total of 22 recordings is collected in the first days of life, while 17 recordings were included from the weeks close to discharge. A detailed overview of each recording is reported in Supplementary Tables 1, 2.

**Figure 3:**
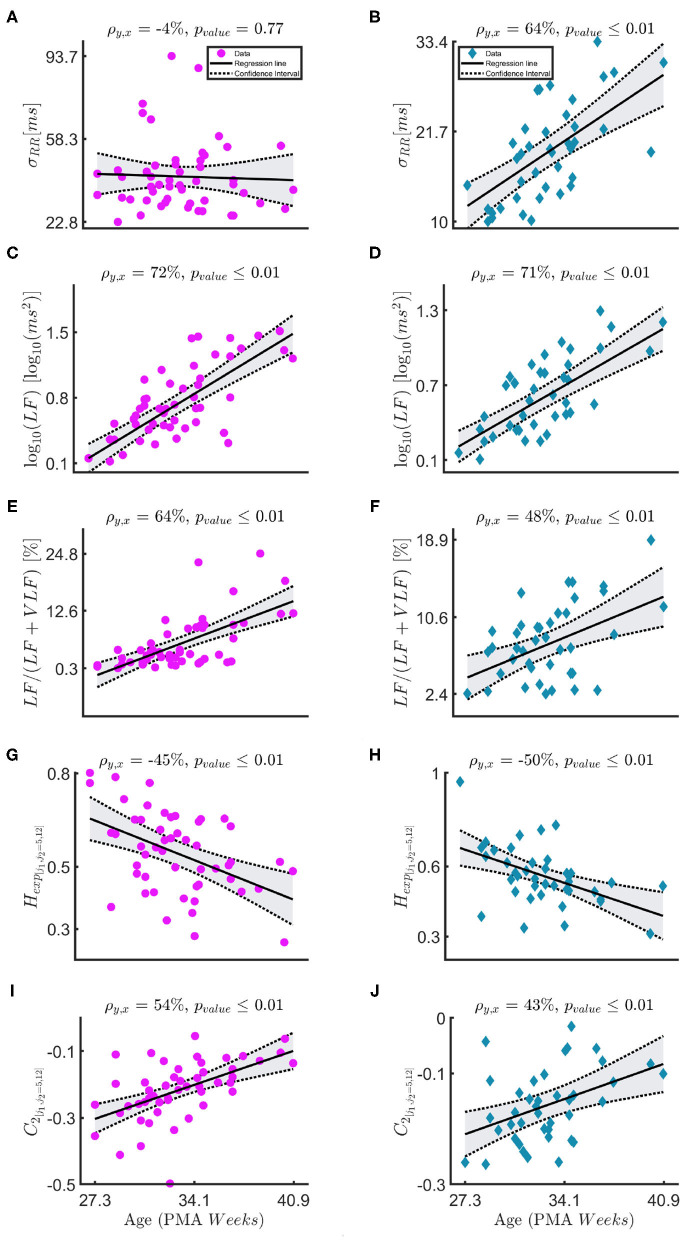
**(A–J)** The figure shows the linear-mixed effect regressions between the post-menstrual age and the following HRV features: the standard deviation of the tachogram σ_*RR*_, the absolute and the relative power in the LF band $\left(\log_{10}\left(LF\right),\frac{LF}{LF+VLF}\right)$, the Hurst exponent *H*_*exp*, [*j*_1_,*j*_2_=5,12]_ and the parameter *C*_2_. The sampling frequencies for the fractal indices is *f*_*s*_ = 8 *Hz*. The left column—magenta circles report the results for the bradycardia epochs, while the right column—indigo diamonds the results for the between-bradycardia epochs. ρ is the correlation coefficient with the associated significance *p*_*value*_.

Figure 3 and Table 3 report the trends in the three different windowing schemes for the following features: the mean μ_*RR*_ and standard deviation of the *HRV* σ_*RR*_, the absolute power in the *LF* band (and its logarithmic transform), the relative $\frac{LF}{LF+VLF}$ power, the Hurst Exponent in the range [*j*_1_,*j*_2_]=[5,12] *H*_*exp*_[*j*_1_,*j*_2_=5,12] and the width of the singularity spectrum in the same range *C*_2_[*j*_1_,*j*_2_ = 5,12]. The overview of all features for all different windowing schemes (*PB*, *BB*, and *WB*) are reported in the Supplementary Tables 3–5. Figure 3 reports the results for the windowing scheme for the within-bradycardia epochs on the left column, while the results for the between-bradycardia epochs are shown on the right column. The power in the LF band and the relative LF power ($\frac{LF}{LF+VLF}$) increase with increasing PMA in both scenarios: in particular, Pearson correlations ρ_*xy*_ are 69% (72% with logarithm transform) and 64% for bradycardia epochs, respectively, while ρ_*xy*_ are 71% (71% with logarithm transform) and 48%, respectively, for between-bradycardia windows. Concerning the post-bradycardia period, the Table 3 shows a Pearson correlation of 69% for the power in LF band and 57% for $\frac{LF}{LF+VLF}$. Results are here reported for the wavelet approach, but the other spectral methodologies exhibit similar trends (see Supplementary Tables 3–5). In addition, the Hurst exponent (derived as the *c*_1_ of the singularity spectrum) decreases with development (ρ_*xy*_ are −45% in the bradycardias scenario, −47% in post-bradycardia scenario, and −50% in the between-bradycardia scenario), while the width of the singularity spectrum (*c*_2_ parameter) increases with increasing PMA (ρ_*xy*_ are 54% in the bradycardia scenario, 45% in the post-bradycardia scenario and 43% in the between-bradycardia scenario). The greatest contrast was found with the variability of the heart-rate, σ_*RR*_. While the standard deviation increases with infants' maturation in the between-bradycardia epochs, the σ_*RR*_ does not increase with age within the bradycardic event. Moreover, it is higher in the bradycardia epochs than in the between-bradycardia scenario (ρ_*bradycardia*_ = −4% vs. ρ_*between*_ = 64% with *p*_*v*_ = 0.77 vs. *p*_*v*_ ≤ 0.01).

**Table 3:** The main temporal, spectral and fractal features are reported for the three windowing schemes (*PB*, *BB*, and *WB*).

**Median (IQR)—PMA weeks**	**** ≤ 32****	****(32−36]****	**> 36**	****ρ(*%*)****
**POST-BRADYCARDIA (PB) GROUP**				
μ_*RR*_	374.65 (366.38–391.36)	377.07 (364.33–393.69)	387.2 (374.98–416.44)	0.39^**^
σ_*RR*_	16.71 (12.02–22.05)	25.5 (21.65–31.1)	28.47 (24.03–32.08)	0.49^**^
*P*(*LF*)_*Wavelet*_	2.14 (0.96–3.68)	4.17 (2.16–8.9)	17.74 (4.94–25.51)	0.69^**^
${\frac{LF}{LF+VLF}}_{Wavelet}$	5.38 (3.45–8.7)	4.9 (3.67–12.17)	14.06 (11.77–18.09)	0.57^**^
*H*_*exp*, [*j*_1_,*j*_2_=5,12]_	0.61 (0.52–0.7)	0.55 (0.45–0.59)	0.5 (0.44–0.56)	−0.47^**^
*C*_2, [*j*_1_,*j*_2_=5,12]_	−0.2 (−0.26 to −0.17)	−0.19 (−0.21 to −0.13)	−0.14 (−0.15 to −0.11)	0.45^**^
**BETWEEN-BRADYCARDIA (BB) GROUP**				
μ_*RR*_	370.51 (359.96–388.36)	377.42 (363.11–389.25)	394.93 (370.01–427.45)	0.47^**^
σ_*RR*_	13.89 (10.97–18.49)	19.81 (15.72–23.82)	29.1 (21.99–30.66)	0.64^**^
*P*(*LF*)_*Wavelet*_	1.3 (0.86–3.38)	4.23 (1.93–6.28)	11.34 (8.4–15)	0.71^**^
${\frac{LF}{LF+VLF}}_{Wavelet}$	6.93 (4.89–8.53)	7.9 (4.63–11.05)	12.57 (8.75–13.95)	0.48^**^
*H*_*exp*, [*j*_1_,*j*_2_=5,12]_	0.6 (0.52–0.68)	0.54 (0.5–0.59)	0.48 (0.45–0.52)	–0.5^**^
*C*_2, [*j*_1_,*j*_2_=5,12]_	−0.19 (−0.23 to −0.14)	−0.17 (−0.2 to −0.14)	−0.09 (−0.12 to −0.08)	0.43^**^
**WITHIN-BRADYCARDIA (WB) GROUP**				
μ_*RR*_	384.9 (369.62–398.91)	384.16 (369.2–397.51)	389.12 (377.65–425.8)	0.37^**^
σ_*RR*_	38.31 (32.22–44.62)	40.61 (32.5–49.81)	35.89 (28.43–40.74)	−0.04^*n*.*s*.^
*P*(*LF*)_*Wavelet*_	2.3 (1.12–4.03)	4.68 (2.8–9.86)	18.66 (5.35–26.46)	0.69^**^
${\frac{LF}{LF+VLF}}_{Wavelet}$	2.57 (1.13–3.97)	3.34 (2.19–8.97)	10.96 (6.69–16.78)	0.64^**^
*H*_*exp*, [*j*_1_,*j*_2_=5,12]_	0.61 (0.49–0.71)	0.55 (0.43–0.62)	0.49 (0.43–0.52)	–0.45^**^
*C*_2, [*j*_1_,*j*_2_=5,12]_	−0.26 (−0.3 to −0.21)	−0.21 (−0.24 to −0.17)	−0.13 (−0.18 to −0.11)	0.54^**^

The results are reported as median (IQR). IQR stands for interquartile range. The fractal indices are reported for f_s_ = 8 Hz. The symbol ρ stands for the Pearson correlation coefficient. The symbol ^**^ represents a significant correlation with p ≤ 0.01 and ^n.s.^ is used to indicate a non-significant correlation.

Table 4 shows the regression results for the linear mixed-effect models, while Table 5 reports the features selected by LASSO. Those two tables report the results for the three different windowing schemes (*PB*, *BB*, *WB*) in three different blocks, while the rows report the results for the different feature groups (temporal, spectral and fractal attributes) and sampling frequencies. The different columns, respectively report the explained variance in the training set ($R_{train}^{2}$), the mean absolute error (*MAE*) and the explained variance in the test set ($R_{test}^{2}$). The best performance is reached for the combination of all features in the *PB* scheme ($R_{train}^{2}=0.75$, *MAE* = 1.83 weeks, $R_{test}^{2}=0.57$) as well as between bradycardias ($R_{train}^{2}=0.68$, *MAE* = 1.56 weeks, $R_{test}^{2}=0.59$). During the bradycardia event (*WB*), the best performance is achieved with the spectral features ($R_{train}^{2}=0.73$, *MAE* = 1.9 weeks, $R_{test}^{2}=0.62$). Table 5 shows that the selected features are the absolute spectral power in *LF* and *VLF* together with *C*_2_ parameter in the range [*j*_1_,*j*_2_] = [5,12] for the first two schemes. For the *WB* scheme, the selected feature is simply the power in *LF* band.

**Table 4:** Linear mixed-effect model performances.

**Feature type**	${\text{R}}_{train}^{2}$	***MAE*(*weeks*)**	${\text{R}}_{test}^{2}$	***P_value_***
**POST-BRADYCARDIA (PB) EPOCHS**				
**Minimum MAE**	0.75 (0.09)	**1.83 (0.41)**	0.57 (0.22)	0 (0)
Temporal features	0.44 (0.28)	2 (0.56)	0.35 (0.19)	0.01 (0.04)
Spectral features	0.74 (0.12)	2.01 (0.42)	0.5 (0.11)	0.00 (0.02)
Fractal features	0.26 (0.1)	2.18 (0.46)	0.43 (0.28)	0.00 (0.04)
**BETWEEN-BRADYCARDIA (BB) EPOCHS**				
**Minimum MAE**	0.68 (0.11)	**1.56 (0.39)**	0.59 (0.16)	0 (0.01)
Temporal features	0.6 (0.33)	2.06 (0.38)	0.44 (0.24)	0.01 (0.03)
Spectral features	0.59 (0.19)	1.93 (0.54)	0.59 (0.15	0 (0.01)
Fractal features	0.34 (0.28)	2.16 (0.53)	0.18 (0.31)	0.06 (0.32)
**WITHIN-BRADYCARDIA (WB) EPOCHS**				
All features	0.72 (0.15)	1.95 (0.33)	0.57 (0.24)	0 (0.01)
*Temporal features*	*0.14 (0.1)*	*2.79 (0.35)*	*0.13 (0.13)*	0.18 (0.35)
**Minimum MAE**	0.73 (0.17)	**1.9 (0.21)**	0.62 (0.21)	0.00 (0.00)
Fractal features	0.4 (0.16)	2.13 (0.56)	0.29 (0.28)	0.02 (0.06)

For each feature set and sampling frequency f_s_, the table shows the median (IQR) over the 20 iterations for $R_{train}^{2}$ on the train set as well as $R_{test}^{2}$, the MAE in weeks on test set and the p_value_ for the F-statistics of the regression. IQR here stands for the difference between 25 and 75% quantiles.

**Table 5:** LASSO selected features for the linear mixed-effect model.

**POST-BRADYCARDIA (**PB**) EPOCHS**			
**Feature type**			
**Minimum MAE**	log_10_(*LF*)_*SPWVD*_	*C*_2, [*j*_1_,*j*_2_=5,12]_	
Spectral	log_10_(*LF*)_*SPWVD*_	log_10_(*LF*)_*Wavelet*_	
Fractal	*C*_2, [*j*_1_,*j*_2_=5,12]_		
**BETWEEN-BRADYCARDIA (**BB**) EPOCHS**			
**Feature type**			
**Minimum MAE**	log_10_(*VLF*)_*Wavelet*_	log_10_(*LF*)_*SPWVD*_	*C*_2, [*j*_1_,*j*_2_=5,12]_
Spectral	log_10_(*LF*)_*SPWVD*_		
Fractal	*C*_2, [*j*_1_,*j*_2_=5,12]_		
**WITHIN-BRADYCARDIA (**WB**) EPOCHS**			
**Feature type**			
All	log_10_(*LF*)_*Wavelet*_	*C*_2, [*j*_1_,*j*_2_=5,12]_	
**Minimum MAE**	log_10_(*LF*)_*Wavelet*_		
Fractal	*H*_*exp*, [*j*_1_,*j*_2_=5,12]_	*C*_2, [*j*_1_,*j*_2_=5,12]_	

For each feature set, the features that have been selected more than 40% of times have been reported. σ_RR_ and μ_RR_ stand for the standard deviation and the mean of HRV. log_10_(LF) and log_10_(VLF) stand for the absolute power in the LF and VLF bands. Results are reported for the Wigner-Ville distribution (SPWD) or the wavelet transform. H_exp, [_j__2_, j_2_]_ and c_2, [_j__2_, j_2_]_ stand for the Hurst exponent and the parameter c_2_ in the range [j_1_, j_2_].

**Figure 4:**
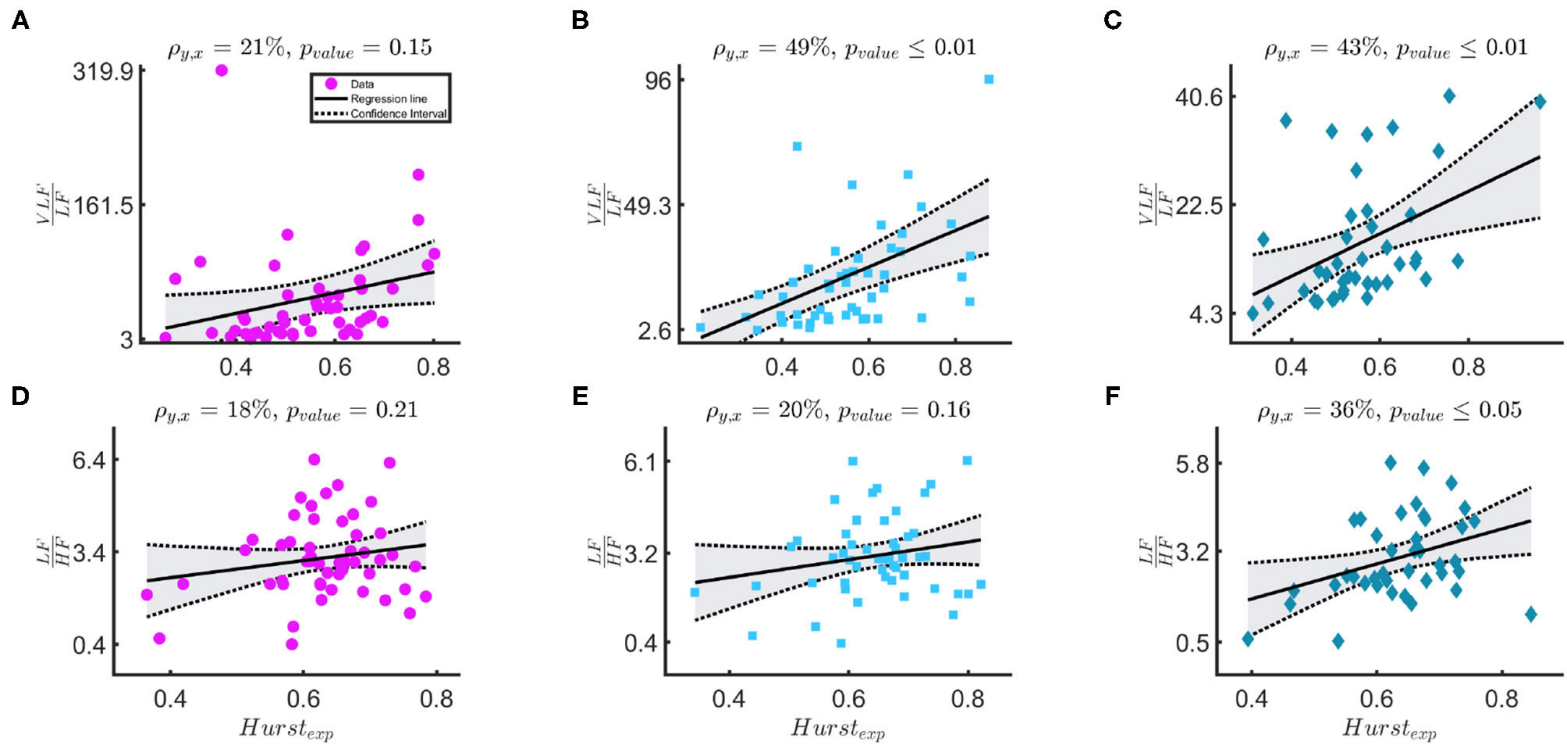
**(A–F)** The figure shows results for the linear-mixed effect regression that models the relationship between *H*_*exp*, [*j*_1_,*j*_2_=5,12]_ and $\frac{VLF}{LF}$ in the first row and between *H*_*exp*, [*j*_1_, *j*_2_ = 3, 12]_ and $\frac{LF}{HF}$ in the second row. The three columns, respectively represents bradycardia epochs (magenta circle data points), post-bradycardia epochs (light-blue squares data points), and between-bradycardia epochs (indigo diamonds data points). ρ is the correlation coefficient of the regression and *p*_*value*_ represents the significance of the correlation.

Figure 4 shows that the relationship of (10) between the *H*_*exp*_ and the ratios $\frac{VLF}{LF}$ and $\frac{LF}{HF}$. The first row shows the relationship between $\frac{VLF}{LF}$ and *H*_*exp*, [*j*_1_,*j*_2_=5,12]_ in the three windowing schemes: *WB* in magenta circles, *PB* in light-blue squares and *BB* in indigo diamonds. The Pearson correlation coefficients are 21, 49, and 43%, respectively. The second row shows the relationship between $\frac{LF}{HF}$ and *H*_*exp*, [*j*_1_, *j*_2_ = 3, 12]_ in the same three schemes. The Pearson correlation coefficients are 18, 20, and 36%, respectively.

## 4. Discussion

This study provides an overview of the autonomic nervous system maturation in preterm infants and aims to estimate the post-menstrual age of the infants based on the HRV. Since the neonatal tachogram is a signal characterized by lack of stationarity and strong slow-wave baseline (Abry et al., 2010; Hoyer et al., 2013; Doret et al., 2015), the current study investigated the maturation of sympathetic and parasympathetic branches with the combination of temporal, spectral and fractal indices. Three main novel findings can be reported. First, Table 4 shows that the maturation of infants can be assessed with different spectral and fractal HRV indices with comparable performances to other maturation models for fetal and preterm development by Hoyer et al. (2013), De Wel et al. (2017), and Lavanga et al. (2017, 2018). Second, Figure 4 reports that the spectral ratio $\frac{VLF}{LF}$ and the Hurst exponent in the range [*j*_1_,*j*_2_]=[5,12] are more correlated than the $\frac{LF}{HF}$ and the Hurst exponent [*j*_1_,*j*_2_]=[3,12]. This might indicate that neonates do not have a sympathovagal balance that relies on the typical interplay between *LF* and *HF* (Abry et al., 2010; Doret et al., 2015). Third, the bradycardias can impact HRV maturational features, especially the most common temporal indices that are used in clinical practice (Javorka et al., 2017), such as the $\frac{LF}{HF}$ ratio and the standard deviation (Tables 3, 4). Additionally, the relationship between spectral ratios and *H*_*exp*_ is strongly reduced in the *WB* scheme, as stressed by Figure 4.

The different age models that were derived in this study can outperform or can be compared to the other developmental models reported in the literature (Hoyer et al., 2013; Lavanga et al., 2018). Specifically, Table 4 highlights the capacity of spectral features to outperform all other features in the PMA estimation in all three windowing schemes. Furthermore, the *LF* power is consistently selected by LASSO for all the different *f*_*s*_ and with any type of windowing scheme. These results are not simply in line with a decrease of $\frac{VLF}{LF}$ by Hoyer et al. (2013), but they are also supported by other clinical findings. Namely, an increase of the short-term variability of the tachogram was found during the first days of life (De Souza Filho et al., 2019) and the absolute *LF* power can discriminate preterm and full-term infants with 84% accuracy (Mulkey et al., 2018). However, the highest performances in the *PB* and *BB* schemes are achieved when the fractal and spectral features are combined, as highlighted in bold in Table 4 and suggested by the concomitant increase of entropy and short-term variability of HRV found by De Souza Filho et al. (2019). Interestingly, the highest performances are also achieved when the between bradycardias epochs are considered (*MAE* = 1.56 weeks), which further reveals a bias effect of bradycardias in the description of autonomic maturation. In line with other studies (Clairambault et al., 1992; Curzi-Dascalova, 1994; Longin et al., 2006; Hoyer et al., 2013), we found that the tachogram mean μ_*RR*_ and its standard deviation σ_*RR*_ increase with maturation together with the absolute power in all investigated frequency bands. These findings also confirm the results by Mulkey et al. (2018), who found a greater discrimination ability of the absolute power than relative indices in the classification between preterm and full-term neonates. The lack of stationarity and the 1/*f* spectrum behavior makes it difficult to describe the autonomic maturation without all the frequency bands in place or the fractal indices *H*_*exp*_ and *c*_2_, which are also involved in the estimation of the development (Table 5).

These findings seem to suggest that the ratio $\frac{LF}{HF}$ is not the most suitable index for the sympathovagal balance and the common HRV frequency bands are suitable for infants. As anticipated, David et al. noticed that the fetal heart-rate has such enhanced slow-wave baseline, which increases the power in the *VLF* band such that both David et al. (2007) and Hoyer et al. (2013) used the ratio $\frac{VLF}{LF}$ as a possible index to describe the sympatho-vagal interplay. This approach is confirmed by the results in Figure 4. As discussed by Abry et al. (2010) and Doret et al. (2015), the spectral ratio is linked to *H*_*exp*_ via (10). The panels suggest that the ANS modulation and its fractal regularity lie in the lower-frequency bands since the *H*_*exp*_ is more correlated with $\frac{VLF}{LF}$. The Pearson correlation coefficients ρ_*xy*_ between $\frac{VLF}{LF}$ and *H* are respectively 21, 49, 43% according to different windowing schemes. These are significantly higher than ρ_*xy*_ between $\frac{LF}{HF}$ and *H*_*exp*_ (18, 20, and 36%). It is important to notice that the *H*_*exp*_ matches the spectral ratio if its estimation range [*j*_1_, *j*_2_] matches the frequency bands with most of the exponential decay in the PSD. In this study, [*j*_1_, *j*_2_ = 5, 12] covers specifically the lower frequency bands. These frequency band's importance is confirmed by the features selected by LASSO (Table 5). In line with Doret et al. (2015), the current findings clearly suggest a redefinition of $\frac{LF}{HF}$ with an extension of frequency bands from the most common adults' range, e.g., *LF* = [0.02−0.15] *Hz* and *HF* = [0.15−1.3] *Hz*. They also highlight the greater prominence of the slower oscillations in the description of premature ANS.

However, the results also highlight the disruptive role of bradycardias in maturation analysis. As anticipated, the best regression results are achieved in the between bradycardia epochs (Table 4), and the relationship between the spectral ratios and the *H*_*exp*_ is disrupted with *WB* windowing (panels with magenta circle in Figure 4). Most importantly, the relationship between the temporal features and maturation is lost, as highlighted by the poor *R*^2^ (Table 4 and panels with magenta circles in Figure 3). In addition, Gee et al. (2017) observed that the *LF* power, the variance and the regularity of the heart-rate increase before bradycardias. The results in Figure 3 and Supplementary Tables 4, 5 support this increase in variance and regularity (as can be easily noticed by the y-axis of σ_*RR*_ or any other features of the left column in Figure 3). This finding clearly implies that the exclusion of bradycardias is fundamental whenever using the standard deviation and the mean of the tachogram to assess the maturation of ANS. Figure 4 also shows that bradycardias annihilate the expected relationship between the spectral ratios and the Hurst exponent. This is further proof that bradycardias disrupt the vagal tone (Porges, 1995), which can distort the PSD power-law in Equation (8). However, Table 4 shows that the autonomic age models within bradycardia can maintain comparable performance to the other two windowing schemes thanks to the spectral features. In particular, Table 5 confirms that the most selected power attribute is the power in *LF* = [0.08 − 0.2] *Hz*, as further proof of the central role of this range in the bradycardic event and ANS maturation (David et al., 2007; Hoyer et al., 2013; Gee et al., 2017). The role of bradycardias might be further investigated via proper conditioning with respiration or SpO_2_ signals. Unfortunately, saturation and respiration data were not available in this dataset and ECG derived respiration does not properly estimate the breathing activity due to the small rib cage of the infants and possible skin stripping (Pereira et al., 2017). As already mentioned, bradycardias can also arise independently from hypoxic or apneic events (Poets et al., 1993). Therefore, this study solely looked at the HR instabilities, but future analysis might comprehend a full cardiovascular assessment to describe the ANS maturation and take into account the influence of the recovery period from the bradycardia spike.

It is also important to highlight some limitations of the current study. One may object to the exclusion of proper sleep-staging in the current analysis, as normally done by Curzi-Dascalova (1994). However, the specific focus on the bradycardia effect strongly limits the number of windows available. On top of that, bradycardias are events normally associated with active sleep (Porges, 1995) and most of the annotated bradycardias in this study were found during states that were not associated with quiet sleep. Similarly, one may also find the number of patients limited, but it was caused by the difficulties in the follow-up. All the included patients had normal developmental outcome at 2 years and the development assessment process is normally characterized by large drop-outs. Concerning the methodology, the different spectral methods (Welch, Wavelet, and Wigner-Ville) show very similar spectral trends, but LASSO more frequently tends to select time-frequency distribution features (Wavelet and Wigner-Ville, Supplementary Tables 4, 5). Although there are studies that claim the superiority of the quadratic time-frequency methods (Orini et al., 2012; Widjaja et al., 2013), the current findings show the wavelet approach would suffice for the spectral analysis. Concerning other sampling frequencies, negligible differences were found and a full discussion is reported in the Supplementary Material. It should also be mentioned that the current study was designed to provide growth charts based on the three principles by Hoyer et al. (2013): increase of variability, pattern formation and increase of complexity. We decided to replace the multiscale entropy with the multifractality due to the non-stationarity of the HRV signals and the relationship between spectral and fractal features (especially the spectral ratio in 10). However, the state space and the increase of complexity could also be monitored by entropy measures, such as the multiscale entropy or the asymmetries of HRV (Porta et al., 2008). In order to have a complete overview of the autonomic changes, future studies should not only analyze the specific frequency bands and the fractal properties of the signal, but they should include changes in the probability densities and the conditional entropy of the signal (Porta et al., 2015, 2017). This might not only provide a universal framework to describe the development of the autonomic nervous system in infants but also a further assessment of the bradycardia impact on the state-space of the tachogram. As also mentioned earlier, a full extension of this analysis should also include respiration signals and arterial blood pressure to provide a complete overview of the cardiovascular regulation of the premature infant (Porta et al., 2006; Montalto et al., 2014).

In a nutshell, the HRV analysis might be a useful tool for development monitoring, but two important factors have to be taken into account. First, the neonatal HRV is characterized by a very-low-frequency tone which requires a redefinition of the different frequency bands to the autonomic stimulation. Second, bradycardias have a disruptive role in the assessment of maturation.

## 5. Conclusion

The present study investigated the maturation of the preterm autonomic nervous system by means of temporal, spectral and fractal features of HRV. Three main findings can be reported. First, infants' maturation can be described by means of multifractal and spectral analysis, which show an increasing trend of *LF* power as well as a decreasing trend of fractal regularity with increasing post-menstrual age. The best obtained performances (*R*^2^ = 0.68 and *MAE* = 1.56 weeks) are obtained as combination of fractal and spectral features and are comparable to other developmental models reported by different authors (Hoyer et al., 2013; De Wel et al., 2017; Lavanga et al., 2017, 2018). Second, this predominance of *LF* and *VLF* bands as well as the lower scales for the multifractal indices suggest that the sympathovagal balance of neonates might not simply be related to the ratio $\frac{LF}{HF}$, but the entire HRV band and the regularity of the tachogram should be included to have a better understanding of the ANS maturation. Third, bradycardias might forcefully increase the variance of the heart rate and disrupt the relationship between autonomic indices and age, especially for commonly used metrics in clinical practice. The PMA estimation models based on novel HRV indices provide a more comprehensive understanding of post-natal autonomic maturation. They can be also considered an alternative automated maturity index to other electrophysiological data analysis for the NICU. This research might be a first step to design personalized therapies or preventive care to preserve infants' development.

## Data Availability Statement

The datasets presented in this article are not readily available because the clinical metadata of patients (such as hospital ID, age, recording time-stamps, pain scales) are subject to the European data-privacy policy. Requests to access the datasets should be directed to mlavanga@esat.kuleuven.be. The authors will try to provide an anonymized version of the dataset in compliance with the privacy policy of the University Hospitals of Leuven, which is the owner of the data.

## Ethics Statement

The studies involving human participants were reviewed and approved by Ethical Committee of University Hospitals Leuven, Belgium. Written informed consent to participate in this study was provided by the participants' legal guardian/next of kin. Written informed consent was obtained from the individual(s), and minor(s)' legal guardian/next of kin, for the publication of any potentially identifiable images or data included in this article.

## Author Contributions

ML wrote the article and conducted the data analysis. EH and JM supported the data analysis. AC and SV supervised the data analysis. KJ and GN conducted the data collection. EH, JM, BB, KJ, EO, GN, SV, and AC reviewed and corrected the manuscript. All authors contributed to the article and approved the submitted version.

## Conflict of Interest

The authors declare that the research was conducted in the absence of any commercial or financial relationships that could be construed as a potential conflict of interest.
