# Supplementary material for: Maturation of the Autonomic Nervous System in Premature Infants: Estimating Development Based on Heart-Rate Variability Analysis
Source: Front Physiol. 2021 Jan 12;11:581250. doi: 10.3389/fphys.2020.581250 (PMC7873975; doi:10.3389/fphys.2020.581250)
Supplement: Supplementary file 1 [file Presentation_1.pdf]

Maturation of the autonomic nervous system in premature infants: estimating development based on heart-rate variability analysis

Mario Lavanga [1,*], Elisabeth Heremans [1], Jonathan Moeyersons [1], Bieke Bollen [2], Katrien Jansen [2], Els Ortibus [2], Gunnar Naulaers [2], Sabine Van Huffel [1], Alexander Caicedo [3]

[1] *Department of Electrical Engineering (ESAT), Division STADIUS, KU Leuven, Leuven, Belgium*
[2] *Department of Development and Regeneration, faculty of Medicine, KU Leuven, Belgium*
[3] *Applied Mathematics and Computer Science, School of Engineering, Science and Technology, Universidad del Rosario, Bogotá, Colombia*

Correspondence*:
Mario Lavanga
mlavanga@esat.kuleuven.be

SUPPLEMENTARY MATERIAL: THE EFFECT OF THE SAMPLING FREQUENCIES

In this section, we reported a full overview of the results based on different sampling frequencies. Based on Table A1, the best regression performance is reached at $f_s = 12\ Hz$ in the PB scheme ($R^2_{train} = 0.75$, $MAE = 1.83\ weeks$, $R^2_{test} = 0.57$) as well as between bradycardias ($R^2_{train} = 0.68$, $MAE = 1.56\ weeks$, $R^2_{test} = 0.59$). During the bradycardia event (WB), the best performance is achieved with the spectral features at $f_s = 6\ Hz$ ($R^2_{train} = 0.73$, $MAE = 1.9$ $weeks$, $R^2_{test} = 0.62$). The LASSO selection is also reported for the different sampling frequencies in Table A2.

The importance of testing different sampling frequencies was highlighted by Bolea et al.,who showed a dependency for nonlinear metrics (such as Sample Entropy) with the resampling frequency of the tachogram (Bolea et al., 2016). Based on their results, Bolea et al. concluded that a resampling frequency correction of nonlinear parameters is needed in cardiovascular applications in order to detect meaningful results (such as experiments with body position changes) (Bolea et al., 2016). The employed fractal indices clearly show a increasing trend with an increasing sampling frequency (Section 2.3.3 and Table A3). However, a correction for the sampling frequency was not implemented. The multifractal properties were investigated with different sampling frequencies and our study shows mild differences in term of regression results and correlation with age (Table A3).

Table A1. Linear mixed-effect model performances for the different sampling frequency f_s.

Post-bradycardia (PB) epochs				
Feature type $\|_{fs}$	R^2_{train}	$MAE(weeks)$	R^2_{test}	P_{value}
All features $\|_{6Hz}$	0.73(0.11)	1.88(0.39)	0.57(0.29)	0(0)
All features $\|_{8Hz}$	0.75(0.1)	1.85(0.3)	0.56(0.23)	0(0)
**All features** $\|_{12Hz}$	0.75(0.09)	**1.83(0.41)**	0.57(0.22)	0(0)
Temporal features	0.44(0.28)	2(0.56)	0.35(0.19)	0.01(0.04)
Spectral features	0.74(0.12)	2.01(0.42)	0.5(0.11)	0(0.02)
Fractal features $\|_{6Hz}$	0.33(0.17)	2.21(0.42)	0.33(0.34)	0.01(0.17)
Fractal features $\|_{8Hz}$	0.35(0.13)	2.31(0.54)	0.21(0.22)	0.07(0.24)
Fractal features $\|_{12Hz}$	0.26(0.1)	2.18(0.46)	0.43(0.28)	0(0.04)
Between-bradycardia (BB) epochs				
Feature type $\|_{fs}$	R^2_{train}	$MAE(weeks)$	R^2_{test}	P_{value}
All features $\|_{6Hz}$	0.55(0.12)	1.82(0.28)	0.57(0.24)	0(0.04)
All features $\|_{8Hz}$	0.6(0.11)	1.81(0.38)	0.55(0.19)	0.01(0.01)
**All features** $\|_{12Hz}$	0.68(0.11)	**1.56(0.39)**	0.59(0.16)	0(0.01)
Temporal features	0.6(0.33)	2.06(0.38)	0.44(0.24)	0.01(0.03)
Spectral features	0.59(0.19)	1.93(0.54)	0.59(0.15	0(0.01)
Fractal features $\|_{6Hz}$	0.3(0.22)	2.57(0.43)	0.15(0.19)	0.16(0.27)
Fractal features $\|_{8Hz}$	0.22(0.26)	2(0.25)	0.24(0.36)	0.09(0.34)
Fractal features $\|_{12Hz}$	0.34(0.28)	2.16(0.53)	0.18(0.31)	0.06(0.32)
Within-Bradycardia (WB) epochs				
Feature type $\|_{fs}$	R^2_{train}	$MAE(weeks)$	R^2_{test}	P_{value}
All features $\|_{6Hz}$	0.73(0.1)	1.97(0.42)	0.58(0.25)	0(0)
All features $\|_{8Hz}$	0.7(0.15)	1.91(0.21)	0.5(0.25)	0(0.01)
All features $\|_{12Hz}$	0.72(0.15)	1.95(0.33)	0.57(0.24)	0(0.01)
Temporal features	*0.14(0.1)*	*2.79(0.35)*	*0.13(0.13)*	0.18(0.35)
**Spectral features**	0.73(0.17)	**1.9(0.21)**	0.62(0.21)	0(0)
Fractal features $\|_{6Hz}$	0.33(0.07)	2.16(0.4)	0.23(0.18)	0.07(0.13)
Fractal features $\|_{8Hz}$	0.36(0.13)	2.03(0.56)	0.43(0.22)	0(0.02)
Fractal features $\|_{12Hz}$	0.4(0.16)	2.13(0.56)	0.29(0.28)	0.02(0.06)

Table A2. LASSO selected features for the linear mixed-effect model for the different sampling frequencies f_s.

	Post-bradycardia (PB) epochs		
Feature type $\|_{fs}$			
All $\|_{6Hz}$	$\log_{10}(LF)_{SPWVD}$	$\log_{10}(LF)_{Wavelet}$	$C_{2,[j_1,j_2=5,12]}$
All $\|_{8Hz}$	$\log_{10}(LF)_{Wavelet}$		
**All** $\|_{12Hz}$	$\log_{10}(LF)_{SPWVD}$	$C_{2,[j_1,j_2=\mathbf{5},12]}$	
Spectral	$\log_{10}(LF)_{SPWVD}$	$\log_{10}(LF)_{Wavelet}$	
Fractal $\|_{6Hz}$	$C_{2,[j_1,j_2=\mathbf{5},12]}$		
Fractal $\|_{8Hz}$	$H_{exp,[j_1,j_2=\mathbf{5},12]}$	$C_{2,[j_1,j_2=\mathbf{5},12]}$	
Fractal $\|_{12Hz}$	$C_{2,[j_1,j_2=\mathbf{5},12]}$		
	Between-bradycardia (BB) epochs		
Feature type $\|_{fs}$			
All $\|_{6Hz}$	μ_{RR}	$\log_{10}(LF)_{SPWVD}$	
All $\|_{8Hz}$	$\log_{10}(LF)_{SPWVD}$		
**All** $\|_{12Hz}$	$\log_{10}(VLF)_{Wavelet}$	$\log_{10}(LF)_{SPWVD}$	$C_{2,[j_1,j_2=\mathbf{5},12]}$
Spectral	$\log_{10}(LF)_{SPWVD}$		
Fractal $\|_{6Hz}$	$H_{exp,[j_1,j_2=\mathbf{5},12]}$	$C_{2,[j_1,j_2=\mathbf{5},12]}$	$H_{exp,[j_1,j_2=3,12]}$
	$C_{2,[j_1,j_2=3,12]}$		
Fractal $\|_{8Hz}$	$H_{exp,[j_1,j_2=\mathbf{5},12]}$		
Fractal $\|_{12Hz}$	$C_{2,[j_1,j_2=\mathbf{5},12]}$		
	Within-Bradycardia (WB) epochs		
Feature type $\|_{fs}$			
All $\|_{6Hz}$	$\log_{10}(LF)_{Wavelet}$	$C_{2,[j_1,j_2=\mathbf{5},12]}$	$C_{2,[j_1,j_2=3,12]}$
All $\|_{8Hz}$	$\log_{10}(LF)_{Wavelet}$	$C_{2,[j_1,j_2=\mathbf{5},12]}$	
All $\|_{12Hz}$	$\log_{10}(LF)_{Wavelet}$	$C_{2,[j_1,j_2=\mathbf{5},12]}$	
**Spectral**	$\log_{10}(LF)_{Wavelet}$		
Fractal $\|_{6Hz}$	$C_{2,[j_1,j_2=\mathbf{5},12]}$	$C_{2,[j_1,j_2=3,12]}$	
Fractal $\|_{8Hz}$	$H_{exp,[j_1,j_2=\mathbf{5},12]}$	$C_{2,[j_1,j_2=\mathbf{5},12]}$	
Fractal $\|_{12Hz}$	$H_{exp,[j_1,j_2=\mathbf{5},12]}$	$C_{2,[j_1,j_2=\mathbf{5},12]}$	

Table A3. The fractal features are reported in three different age categories and for the investigated sampling frequencies $f_s = [6, 8, 12]\ Hz$. The results are reported as median(IQR) for the between-bradycardia and bradycardia periods. IQR stands for *interquartile range*. The symbol ρ stands for the Pearson correlation coefficient. The symbol ** represents a significant correlation with $p \leq 0.01$, and * is used for a significant correlation with $p \leq 0.05$. $^{n.s.}$ is used to indicate a non-significant correlation.

Median(IQR) - PMA weeks	≤ 32	$(32-36]$	> 36	$\rho(\%)$
	Fractal features in the PB group, $f_s = 6\ Hz$			
$H_{exp,[j_1,j_2=5,12]}$	0.53(0.46-0.67)	0.49(0.41-0.58)	0.45(0.36-0.5)	-0.45**
$C_{2,[j_1,j_2=5,12]}$	-0.2(-0.25 - -0.18)	-0.19(-0.22 - -0.15)	-0.13(-0.17 - -0.1)	0.57**
$H_{exp,[j_1,j_2=3,12]}$	0.65(0.56-0.71)	0.6(0.55-0.68)	0.56(0.53-0.6)	-0.4**
$C_{2,[j_1,j_2=3,12]}$	-0.17(-0.18 - -0.13)	-0.13(-0.16 - -0.11)	-0.09(-0.12 - -0.08)	0.41**
	Fractal features in the PB group, $f_s = 8\ Hz$			
$H_{exp,[j_1,j_2=5,12]}$	0.61(0.52-0.7)	0.55(0.45-0.59)	0.5(0.44-0.56)	-0.47**
$C_{2,[j_1,j_2=5,12]}$	-0.2(-0.26 - -0.17)	-0.19(-0.21 - -0.13)	-0.14(-0.15 - -0.11)	0.45**
$H_{exp,[j_1,j_2=3,12]}$	0.67(0.6-0.71)	0.66(0.59-0.69)	0.62(0.58-0.65)	-0.33*
$C_{2,[j_1,j_2=3,12]}$	-0.14(-0.16 - -0.1)	-0.11(-0.14 - -0.08)	-0.09(-0.11 - -0.09)	0.2$^{n.s.}$
	Fractal features in the PB group, $f_s = 12\ Hz$			
$H_{exp,[j_1,j_2=5,12]}$	0.62(0.52-0.7)	0.56(0.49-0.64)	0.53(0.44-0.54)	-0.45**
$C_{2,[j_1,j_2=5,12]}$	-0.2(-0.23 - -0.16)	-0.17(-0.19 - -0.15)	-0.11(-0.13 - -0.1)	0.57**
$H_{exp,[j_1,j_2=3,12]}$	0.67(0.61-0.73)	0.64(0.61-0.71)	0.62(0.6-0.63)	-0.33*
$C_{2,[j_1,j_2=3,12]}$	-0.13(-0.15 - -0.11)	-0.11(-0.13 - -0.09)	-0.09(-0.11 - -0.08)	0.22$^{n.s.}$

Median(IQR) - PMA weeks	≤ 32	$(32-36]$	> 36	$\rho(\%)$
	Fractal features in the BB group, $f_s = 6\ Hz$			
$H_{exp,[j_1,j_2=5,12]}$	0.55(0.45-0.65)	0.52(0.43-0.55)	0.45(0.4-0.48)	-0.43**
$C_{2,[j_1,j_2=5,12]}$	-0.19(-0.24 - -0.16)	-0.17(-0.2 - -0.13)	-0.11(-0.12 - -0.09)	0.52**
$H_{exp,[j_1,j_2=3,12]}$	0.65(0.56-0.69)	0.61(0.57-0.67)	0.55(0.52-0.59)	-0.39*
$C_{2,[j_1,j_2=3,12]}$	-0.15(-0.17 - -0.12)	-0.13(-0.15 - -0.1)	-0.08(-0.1 - -0.06)	0.39*
	Fractal features in the BB group, $f_s = 8\ Hz$			
$H_{exp,[j_1,j_2=5,12]}$	0.6(0.52-0.68)	0.54(0.5-0.59)	0.48(0.45-0.52)	-0.5**
$C_{2,[j_1,j_2=5,12]}$	-0.19(-0.23 - -0.14)	-0.17(-0.2 - -0.14)	-0.09(-0.12 - -0.08)	0.43**
$H_{exp,[j_1,j_2=3,12]}$	0.68(0.61-0.73)	0.65(0.6-0.67)	0.6(0.55-0.62)	-0.36*
$C_{2,[j_1,j_2=3,12]}$	-0.12(-0.15 - -0.1)	-0.12(-0.14 - -0.1)	-0.08(-0.09 - -0.05)	0.23$^{n.s.}$
	Fractal features in the BB group, $f_s = 12\ Hz$			
$H_{exp,[j_1,j_2=5,12]}$	0.62(0.52-0.68)	0.57(0.52-0.63)	0.52(0.48-0.53)	-0.43**
$C_{2,[j_1,j_2=5,12]}$	-0.18(-0.23 - -0.16)	-0.15(-0.19 - -0.12)	-0.09(-0.11 - -0.08)	0.53**
$H_{exp,[j_1,j_2=3,12]}$	0.68(0.6-0.71)	0.64(0.6-0.69)	0.59(0.54-0.64)	-0.31$^{n.s.}$
$C_{2,[j_1,j_2=3,12]}$	-0.12(-0.14 - -0.11)	-0.11(-0.12 - -0.1)	-0.08(-0.1 - -0.06)	0.26$^{n.s.}$

Median(IQR) - PMA weeks	≤ 32	$(32-36]$	> 36	$\rho(\%)$
	Fractal features in the WB period, $f_s = 6\ Hz$			
$H_{exp,[j_1,j_2=5,12]}$	0.52(0.42-0.7)	0.48(0.39-0.52)	0.43(0.4-0.46)	-0.36**
$C_{2,[j_1,j_2=5,12]}$	-0.23(-0.29 - -0.2)	-0.21(-0.24 - -0.17)	-0.14(-0.18 - -0.12)	0.55**
$H_{exp,[j_1,j_2=3,12]}$	0.62(0.58-0.67)	0.6(0.53-0.63)	0.57(0.5-0.6)	-0.31*
$C_{2,[j_1,j_2=3,12]}$	-0.19(-0.23 - -0.15)	-0.16(-0.19 - -0.14)	-0.1(-0.12 - -0.09)	0.48**
	Fractal features in the WB period, $f_s = 8\ Hz$			
$H_{exp,[j_1,j_2=5,12]}$	0.61(0.49-0.71)	0.55(0.43-0.62)	0.49(0.43-0.52)	-0.45**
$C_{2,[j_1,j_2=5,12]}$	-0.26(-0.3 - -0.21)	-0.21(-0.24 - -0.17)	-0.13(-0.18 - -0.11)	0.54**
$H_{exp,[j_1,j_2=3,12]}$	0.66(0.62-0.71)	0.64(0.58-0.68)	0.61(0.58-0.62)	-0.36**
$C_{2,[j_1,j_2=3,12]}$	-0.15(-0.2 - -0.12)	-0.14(-0.17 - -0.11)	-0.11(-0.12 - -0.09)	0.31*
	Fractal features in the WB period, $f_s = 12\ Hz$			
$H_{exp,[j_1,j_2=5,12]}$	0.58(0.5-0.68)	0.56(0.5-0.6)	0.5(0.49-0.53)	-0.36**
$C_{2,[j_1,j_2=5,12]}$	-0.22(-0.28 - -0.19)	-0.19(-0.22 - -0.17)	-0.12(-0.14 - -0.1)	0.58**
$H_{exp,[j_1,j_2=3,12]}$	0.64(0.61-0.68)	0.65(0.61-0.68)	0.63(0.6-0.65)	-0.18$^{n.s.}$
$C_{2,[j_1,j_2=3,12]}$	-0.16(-0.18 - -0.11)	-0.13(-0.16 - -0.11)	-0.11(-0.11 - -0.09)	0.32*

SUPPLEMENTARY MATERIAL: OVERVIEW TABLES

Table S1. A detail overview of the recordings of the included patients: the study of each patient ID (PAT_{ID}), the serial number that indicates the recording for each patient (REC_{ID}), the duration of the recording in minutes ($Duration_{Rec}$), average duration of the annotated bradycardias in s for the recording ($Duration_{WB}$),the number of the annotated bradycardias in the recording ($Number_{WB}$), the average RR amplitude during the bradycardia in ms (RR_{WB}), the sampling frequency of the ECG used to derive the tachogram in Hz ($F_{S,ECG}$), postmenstrual age in weeks (PMA) and gestational age in weeks (GA). The last three rows represent the number of recording for each age subgroups (below 32 PMA weeks, between 32 and 36 weeks and above 36 weeks). Blank spaces indicates that bradycardias were not annotated according to the definition in Section **??**.

PAT_{ID}	REC_{ID}	$Duration_{Rec}$	$Duration_{WB}$	$Number_{WB}$	RR_{WB}	$F_{S,ECG}$	PMA	GA
18	3	210.438				250	34.57	25
22	1	296.328	20.625	8	703	250	28.71	25.43
22	2	175.143	27.417	1	808	250	34.29	25.43
174	1	262.792	15.306	3	645	500	32.29	31.43
174	2	272.96	28.583	2	460	250	34.29	31.43
32	2	65.065				250	36	32
32	3	133.833				250	37.43	32
33	1	57.332	9.667	1	610	500	27.29	25.71
33	2	184.832	27.639	9	540	500	29	25.71
33	3	120.942	11.75	1	730	500	31.14	25.71
33	4	264.74	25.156	16	572	500	32.71	25.71
33	5	228.619	17.319	6	603	500	34.71	25.71
34	1	196.621	23.292	12	607	500	27.29	25.71
34	2	136.526	31.208	6	599	500	29	25.71
34	3	128.192	11.833	1	633	500	31	25.71
34	4	251.589	22.893	7	598	500	32.71	25.71
34	5	146.753	18.463	9	590	500	34.71	25.71
34	6	539.988	19.683	10	666	250	40	25.71
35	2	221.486	6.667	1	771	250	34.14	30.43
35	3	251.707	6.583	1	631	250	35.71	30.43
35	4	446.989				250	36.57	30.43
37	1	243.658	23.515	11	612	500	31	30.29
37	2	223.356	14.048	21	630	500	33	30.29
37	3	577.278	29.842	61	524	250	36.71	30.29
38	1	258.396	18.25	5	814	500	31	30.29
38	2	251.453	4.667	2	664	500	33	30.29
38	3	565.986	27.258	59	564	250	36.71	30.29
40	1	100.2				250	31.43	30.29
40	2	495.688				250	36	30.29
44	1	303.978	14.033	5	606	500	29.57	24.57
44	2	195.789	13.1	5	607	500	31.57	24.57
44	3	146.025	21.083	1	577	500	34.57	24.57
44	4	81.593				500	37.71	24.57
60	2	213.171				500	34	31.89
60	3	472.993	5.083	1	592	250	36.29	31.89

Table S2. Continuation of Table S1.

PAT_{ID}	REC_{ID}	$Duration_{Rec}$	$Duration_{WB}$	$Number_{WB}$	$RR_{WB})$	$F_{S,ECG}$	PMA	GA
72	1	149.482	24.292	6	728	500	30.71	30
72	2	234.721	12.167	4	624	500	32.29	30
72	3	54.044				500	34	30
72	4	138.394				500	36	30
72	5	379.979	6.75	1	747	500	37.43	30
75	1	213.293	25.15	5	599	500	31.29	28.86
75	2	149.963				500	33.29	28.86
106	1	272.81	18.021	4	619	500	31.71	26.29
106	2	211.19	29.444	3	560	500	33.57	26.29
106	3	103.571				250	36.71	26.2857
79	1	271.892				500	30.29	29.86
79	2	130.792				500	33.71	29.86
79	4	129.625				500	36.71	29.86
79	5	137.285				500	40.57	29.86
88	2	175.725	24.722	3	716	500	30.43	26.71
88	3	236.161	18.604	12	613	500	32.14	26.71
88	4	60.208				500	35.29	26.71
88	5	142.931				500	37.14	26.71
90	1	241.475	14.833	4	564	500	28.71	25.29
90	2	184.988	22.6	5	615	500	30.43	25.29
90	4	68.238				500	34.57	25.29
90	5	184.693				500	38.57	25.29
159	1	255.615				500	32.57	29.57
159	2	209.44	13.317	5	763	500	34.43	29.57
159	3	98.794				500	36.57	29.57
160	1	165				500	32.71	29.57
160	2	92.985				500	36.71	29.57
160	3	170.431	11.583	3	788	500	40.86	29.57
171	1	125.431				500	32.14	29.29
171	2	88.658	32.833	4	514	500	33.71	29.29
171	3	217.708				500	40.29	29.29
133	1	193.583	12	3	570	500	30.71	27.71
133	2	186.324	39.083	1	597	500	32.43	27.71
133	3	63.25	7.583	2	709	500	34.43	27.71
56	2	107.964	20.542	2	598	250	33.14	30.57
57	1	173.315				500	30.86	30.57
57	2	292.147	7.472	3	604	500	33.14	30.57
19	1	246.489	24.783	5	625	250	32	30
19	2	143.161	26.667	7	565	250	34.57	30

Table S3. The main temporal, spectral and fractal features are reported for the post-bradycardia periods. The results are reported as median(IQR). IQR stands for *interquartile range*. The fractal indices are reported for $f_s = 8\ Hz$. The symbol ρ stands for the Pearson correlation coefficient. The symbol ** represents a significant correlation with $p \leq 0.01$, and * is used for a significant correlation with $p \leq 0.05$. $^{n.s.}$ is used to indicate a non-significant correlation.

Median(IQR) - PMA weeks	≤ 32	$(32-36]$	> 36	$\rho(\%)$
Temporal features in the post-bradycardia (PB) group				
μ_{RR}	374.65(366.38-391.36)	377.07(364.33-393.69)	387.2(374.98-416.44)	0.39**
σ_{RR}	16.71(12.02-22.05)	25.5(21.65-31.1)	28.47(24.03-32.08)	0.49**
Spectral features in the post-bradycardia (PB) group				
$P(VLF)_{Welch}$	106.24(63.14-156.22)	250.27(180.65-408.05)	287.59(219.04-454.15)	0.38**
$P(VLF)_{SPWD}$	643.16(434.61-1029.4)	1787.69(1081.86-2475.95)	2013.8(1252.61-2653.49)	0.39**
$P(VLF)_{Wavelet}$	36.25(19.22-56.58)	82.2(46.77-119.8)	89.58(62.29-139.78)	0.34*
$P(LF)_{Welch}$	10.91(5.35-16.13)	28.98(13.41-48.24)	50.5(19.82-67.56)	0.63**
$P(LF)_{SPWD}$	70.65(29-94.04)	141.99(84.7-219.13)	450.23(119.78-574.82)	0.69**
$P(LF)_{Wavelet}$	2.14(0.96-3.68)	4.17(2.16-8.9)	17.74(4.94-25.51)	0.69**
$P(HF)_{Welch}$	7.85(3.88-9.53)	9.99(6.08-13.7)	11.84(9.28-24.15)	0.22$^{n.s.}$
$P(HF)_{SPWD}$	38.05(22.98-68.8)	76.75(42.2-106.64)	124.07(87.74-210.87)	0.39**
$P(HF)_{Wavelet}$	0.69(0.36-1.3)	1.61(0.67-2.28)	3.57(1.55-6.4)	0.61**
$\frac{VLF}{LF}_{Welch}$	12.22(7.92-24.59)	9.9(5.8-18.72)	5.45(4.72-8.03)	0.06$^{n.s.}$
$\frac{VLF}{LF}_{SPWD}$	10.76(7.7-13.27)	7.68(4.98-15.57)	4.5(3.11-4.82)	-0.08$^{n.s.}$
$\frac{VLF}{LF}_{Wavelet}$	20.17(12.68-34.29)	19.6(7.22-26.37)	6.98(4.53-10.08)	-0.36**
$\frac{LF}{HF}_{Welch}$	1.47(1.05-1.96)	2.33(1.54-3.36)	3.91(1.9-4.94)	0.56**
$\frac{LF}{HF}_{SPWD}$	1.38(1.14-1.79)	1.91(1.71-2.74)	3.2(1.46-3.81)	0.57**
$\frac{LF}{HF}_{wavelet}$	2.27(1.87-3.02)	3.2(2.76-3.75)	4.56(3.04-5.24)	0.48**
$\frac{LF}{LF+HF}_{Welch}$	59.45(48.87-65.64)	69.97(60.1-76.75)	79.55(65.51-83.17)	0.45**
$\frac{LF}{LF+HF}_{SPWD}$	57.89(53.12-63.96)	65.63(63.1-73.15)	76.13(59.34-79.22)	0.48**
$\frac{LF}{LF+HF}_{Wavelet}$	69.44(65.16-74.67)	76.18(73.35-78.44)	81.96(75.27-83.65)	0.37**
$\frac{LF}{LF+VLF}_{Welch}$	9.11(5.32-12.28)	9.17(5.2-15.27)	15.99(13.78-17.47)	0.48**
$\frac{LF}{LF+VLF}_{SPWD}$	9.14(7.5-11.74)	11.53(6.04-16.74)	19.79(17.18-21.91)	0.56**
$\frac{LF}{LF+VLF}_{Wavelet}$	5.38(3.45-8.7)	4.9(3.67-12.17)	14.06(11.77-18.09)	0.57**
Fractal features in the post-bradycardia (PB) group				
$H_{exp,[j_1,j_2=5,12]}$	0.61(0.52-0.7)	0.55(0.45-0.59)	0.5(0.44-0.56)	-0.47**
$C_{2,[j_1,j_2=5,12]}$	-0.2(-0.26 - -0.17)	-0.19(-0.21 - -0.13)	-0.14(-0.15 - -0.11)	0.45**
$H_{exp,[j_1,j_2=3,12]}$	0.67(0.6-0.71)	0.66(0.59-0.69)	0.62(0.58-0.65)	-0.33*
$C_{2,[j_1,j_2=3,12]}$	-0.14(-0.16 - -0.1)	-0.11(-0.14 - -0.08)	-0.09(-0.11 - -0.09)	0.2$^{n.s.}$

Table S4. The main temporal, spectral and fractal features are reported for the between-bradycardias (BB) periods. The results are reported as median(IQR). IQR stands for *interquartile range*. The fractal indices are reported for $f_s = 8\ Hz$. The symbol ρ stands for the Pearson correlation coefficient. The symbol ** represents a significant correlation with $p \leq 0.01$, and * is used for a significant correlation with $p \leq 0.05$. $^{n.s.}$ is used to indicate a non-significant correlation.

Temporal features in the between-bradycardias (BB) group				
Median(IQR) - PMA weeks	≤ 32	$(32-36]$	> 36	$\rho(\%)$
Fractal features in the three age groups				
μ_{RR}	370.51(359.96-388.36)	377.42(363.11-389.25)	394.93(370.01-427.45)	0.47**
σ_{RR}	13.89(10.97-18.49)	19.81(15.72-23.82)	29.1(21.99-30.66)	0.64**
Spectral features in the between-bradycardias (BB) group				
$P(VLF)_{Welch}$	68.99(46.83-128.31)	156.67(100.26-217.63)	320.47(184.27-388.76)	0.54**
$P(VLF)_{SPWD}$	358.71(321.19-673.85)	991.87(498.71-1346.25)	2332.8(901.57-2915.13)	0.58**
$P(VLF)_{Wavelet}$	19.23(16.42-40.32)	58.93(35.74-75.05)	108(55.74-147.03)	0.63**
$P(LF)_{Welch}$	8.27(3.78-13.34)	14.99(5.95-25.28)	35.69(26.87-49.37)	0.66**
$P(LF)_{SPWD}$	46.5(25.63-88.72)	106.67(56.24-154.71)	273.11(213.86-373.31)	0.73**
$P(LF)_{Wavelet}$	1.3(0.86-3.38)	4.23(1.93-6.28)	11.34(8.4-15)	0.71**
$P(HF)_{Welch}$	4.85(3.47-6.9)	5.2(4.06-8.12)	11.55(6.11-13.56)	$0.17^{n.s.}$
$P(HF)_{SPWD}$	24.07(19.35-49.89)	50.8(25.85-85.57)	103.73(63.59-129.27)	$0.24^{n.s.}$
$P(HF)_{Wavelet}$	0.45(0.36-1.06)	0.91(0.53-2.2)	2.79(2.09-4.65)	0.62**
$\frac{VLF}{LF}_{Welch}$	9.4(7.9-13.65)	9.22(5.58-18.69)	5.29(4.7-8.4)	$-0.2^{n.s.}$
$\frac{VLF}{LF}_{SPWD}$	7.86(6.19-10.78)	7.5(5.3-13.48)	4.02(3.45-6.87)	$-0.14^{n.s.}$
$\frac{VLF}{LF}_{Wavelet}$	13.42(10.75-19.77)	11.73(8.1-21.36)	7(6.17-10.44)	$-0.3^{n.s.}$
$\frac{LF}{HF}_{Welch}$	1.42(0.75-2.16)	2.19(1.8-3.09)	3.78(2.39-4.23)	0.57**
$\frac{LF}{HF}_{SPWD}$	1.45(0.98-1.6)	1.87(1.61-2.18)	2.8(1.61-3.25)	0.52**
$\frac{LF}{HF}_{Wavelet}$	2.32(1.8-2.84)	3.2(2.39-4.11)	3.67(2.47-4.6)	0.33*
$\frac{LF}{LF+HF}_{Welch}$	58.67(42.64-67.61)	68.69(64.25-75.48)	78.97(70.53-80.88)	0.56**
$\frac{LF}{LF+HF}_{SPWD}$	59.11(49.57-61.62)	65.19(61.76-68.51)	73.67(61.65-76.48)	0.47**
$\frac{LF}{LF+HF}_{Wavelet}$	69.86(64.13-73.93)	76.21(70.43-80.43)	78.39(71.21-82.13)	0.33*
$\frac{LF}{LF+VLF}_{Welch}$	9.71(6.91-11.29)	9.87(5.18-15.21)	16.08(10.77-17.58)	0.42**
$\frac{LF}{LF+VLF}_{SPWD}$	11.29(8.24-13.6)	11.67(6.91-15.29)	19.55(12.12-21.93)	0.44**
$\frac{LF}{LF+VLF}_{Wavelet}$	6.93(4.89-8.53)	7.9(4.63-11.05)	12.57(8.75-13.95)	0.48**
Fractal features in the between-bradycardias (BB) group				
$H_{exp,[j_1,j_2=5,12]}$	0.6(0.52-0.68)	0.54(0.5-0.59)	0.48(0.45-0.52)	-0.5**
$C_{2,[j_1,j_2=5,12]}$	-0.19(-0.23 - -0.14)	-0.17(-0.2 - -0.14)	-0.09(-0.12 - -0.08)	0.43**
$H_{exp,[j_1,j_2=3,12]}$	0.68(0.61-0.73)	0.65(0.6-0.67)	0.6(0.55-0.62)	-0.36*
$C_{2,[j_1,j_2=3,12]}$	-0.12(-0.15 - -0.1)	-0.12(-0.14 - -0.1)	-0.08(-0.09 - -0.05)	$0.23^{n.s.}$

Table S5. The main temporal, spectral and fractal features are reported for the within-bradycardia (WB) periods. The results are reported as median(IQR). IQR stands for *interquartile range*. The temporal and spectral indices are reported for $f_s = 6\ Hz$ and the fractal index is reported for $f_s = 8\ Hz$. The symbol ρ stands for the Pearson correlation coefficient. The symbol ** represents a significant correlation with $p \leq 0.01$, and * is used for a significant correlation with $p \leq 0.05$. $^{n.s.}$ is used to indicate a non-significant correlation.

Temporal features in the within-bradycardia (WB) group.				
Median(IQR) - PMA weeks	≤ 32	$(32-36]$	> 36	$\rho(\%)$
Fractal features in the three age groups				
μ_{RR}	384.9(369.62-398.91)	384.16(369.2-397.51)	389.12(377.65-425.8)	0.37**
σ_{RR}	38.31(32.22-44.62)	40.61(32.5-49.81)	35.89(28.43-40.74)	-0.04$^{n.s.}$
Spectral features in the the within-bradycardia (WB) group.				
$P(VLF)_{Welch}$	167.75(101.36-282.21)	312.85(213.81-435.07)	300.05(226.89-457.86)	0.28*
$P(VLF)_{SPWD}$	1109.26(838.07-1615.84)	2025.93(1381.79-3909.12)	2168.01(1229.65-3824.98)	0.31*
$P(VLF)_{Wavelet}$	82.66(46.46-169.3)	122.69(69.22-230.91)	128.03(77.63-164.54)	0.1$^{n.s.}$
$P(LF)_{Welch}$	13(6.4-18.71)	31.09(15.62-52.49)	49.95(20.66-73.54)	0.59**
$P(LF)_{SPWD}$	102.62(68.87-185.63)	216.65(136.5-351.6)	502.28(152.3-734.74)	0.65**
$P(LF)_{Wavelet}$	2.3(1.12-4.03)	4.68(2.8-9.86)	18.66(5.35-26.46)	0.69**
$P(HF)_{Welch}$	7.73(4.31-10.01)	10.63(7.49-14.56)	12.21(9.75-25.52)	0.19$^{n.s.}$
$P(HF)_{SPWD}$	78.85(56.13-117.78)	106.08(78.08-182.35)	148.54(118.65-225.94)	0.3*
$P(HF)_{Wavelet}$	0.6(0.4-1.34)	1.49(0.74-2.57)	3.75(1.58-6.34)	0.59**
$\frac{VLF}{LF}_{Welch}$	13.25(9.99-25.23)	9.86(5.99-19.16)	5.62(4.53-7.99)	0.01$^{n.s.}$
$\frac{VLF}{LF}_{SPWD}$	8.3(6.56-12.13)	8.85(4.78-12.81)	4.18(2.93-4.69)	-0.08$^{n.s.}$
$\frac{VLF}{LF}_{Wavelet}$	43.62(24.21-96.77)	30.2(10.15-56.67)	9.21(4.96-13.94)	-0.47**
$\frac{LF}{HF}_{Welch}$	1.59(1.2-2.07)	2.28(1.69-3.51)	3.86(1.85-4.99)	0.52**
$\frac{LF}{HF}_{SPWD}$	1.3(1.19-1.51)	1.8(1.48-2.26)	2.89(1.35-3.25)	0.57**
$\frac{LF}{HF}_{Wavelet}$	2.36(1.89-2.99)	3.23(2.95-3.84)	4.67(3.02-5.3)	0.48**
$\frac{LF}{LF+HF}_{Welch}$	61.12(53.47-66.49)	69.5(62.38-77.32)	79.32(64.9-83.31)	0.41**
$\frac{LF}{LF+HF}_{SPWD}$	56.49(54.27-60.15)	64.06(59.6-69.35)	74.27(57.44-76.58)	0.49**
$\frac{LF}{LF+HF}_{Wavelet}$	70.22(65.4-74.94)	76.37(73.75-78.74)	82.34(75.1-84.11)	0.37**
$\frac{LF}{LF+VLF}_{Welch}$	7.28(4.92-9.1)	9.2(4.99-14.83)	15.38(14.34-18.08)	0.55**
$\frac{LF}{LF+VLF}_{SPWD}$	10.86(8.03-13.38)	10.86(7.2-17.11)	19.63(18.13-23.51)	0.52**
$\frac{LF}{LF+VLF}_{Wavelet}$	2.57(1.13-3.97)	3.34(2.19-8.97)	10.96(6.69-16.78)	0.64**
Fractal features in the within-bradycardia (WB) group.				
$H_{exp,[j_1,j_2=5,12]}$	0.61(0.49-0.71)	0.55(0.43-0.62)	0.49(0.43-0.52)	-0.45**
$C_{2,[j_1,j_2=5,12]}$	-0.26(-0.3- -0.21)	-0.21(-0.24- -0.17)	-0.13(-0.18- -0.11)	0.54**
$H_{exp,[j_1,j_2=3,12]}$	0.66(0.62-0.71)	0.64(0.58-0.68)	0.61(0.58-0.62)	-0.36**
$C_{2,[j_1,j_2=3,12]}$	-0.15(-0.2- -0.12)	-0.14(-0.17- -0.11)	-0.11(-0.12- -0.09)	0.31*

Table S6. A detail overview of the average number of beats for the different states period defined in this study: the average duration of the annotated bradycardias (Braydcardia) and the average durations of the between-bradycardia window (Duration BB), the post-bradycardia window (Duration PB) and the within-bradycardia window (Duration WB). Blank spaces indicates that bradycardias were not annotated according to the definition in Section **??** or windows were not available (e.g. there were not enough 10 minutes window between bradycardia events).

PAT_{ID}	REC_{ID}	Bradycardia [$\#Beats$]	Duration BB [$\#Beats$]	Duration PB $\#Beats$	Duration WB [$\#Beats$]
18	3				
22	1	33	1546	1574	1565
22	2	35		1371	1340
174	1	28	1544	1475	1463
174	2	63	1568	1379	1367
32	2				
32	3				
33	1	16		1634	1621
33	2	60	1657	1643	1627
33	3	16		1545	1528
33	4	49	1650	1638	1622
33	5	36	1568	1623	1601
34	1	40	1702	1679	1658
34	2	51	1585	1615	1589
34	3	19		1526	1512
34	4	40	1565	1603	1574
34	5	34	1532	1522	1508
34	6	35	1421	1437	1423
35	2	9		1461	1452
35	3	10		1525	1516
35	4				
37	1	48	1557	1628	1612
37	2	27	1706	1740	1728
37	3	66	1642	1686	1677
38	1	26	1551	1582	1556
38	2	8	1732	1699	1686
38	3	55	1624	1649	1630
40	1				
40	2				
44	1	31	1645	1666	1656
44	2	24	1666	1638	1622
44	3	38		1536	1520
44	4				
60	2				
60	3	8		1731	1710

Table S7. Continuation of Table S6.

PAT$_{ID}$	REC$_{ID}$	Duration Bradycardia [$\#Beats$]	Duration BB [$\#Beats$]	Duration PB $\#Beats$	Duration WB [$\#Beats$]
72	1	34	1438	1369	1357
72	2	21	1596	1559	1544
72	3				
72	4				
72	5	9		1318	1311
75	1	43	1590	1511	1491
75	2				
106	1	34	1716	1666	1651
106	2	52	1689	1588	1587
106	3				
79	1				
79	2				
79	4				
79	5				
88	2	36	1581	1621	1586
88	3	32	1685	1629	1603
88	4				
88	5				
90	1	35	1649	1660	1653
90	2	42		1613	1580
90	4				
90	5				
159	1				
159	2	18	1549	1552	1532
159	3				
160	1				
160	2				
160	3	15	1392	1409	1403
171	1				
171	2	74	1567	1585	1577
171	3				
133	1	25	1739	1689	1679
133	2	75		1861	1818
133	3	11	1674	1645	1637
56	2	38			
57	1				
57	2	12	1584	1599	1588
19	1	44	1659	1552	1523
19	2	50	1606	1544	1533
